# The Safety, Tolerability, and Effects on the Systemic Inflammatory Response and Renal Function of the Human Chorionic Gonadotropin Hormone-Derivative EA-230 Following On-Pump Cardiac Surgery (The EASI Study): Protocol for a Randomized, Double-Blind, Placebo-Controlled Phase 2 Study

**DOI:** 10.2196/11441

**Published:** 2019-02-06

**Authors:** Roger van Groenendael, Remi Beunders, Jan Hofland, Wim J Morshuis, Matthijs Kox, Lucas T van Eijk, Peter Pickkers

**Affiliations:** 1 Department of Intensive Care Medicine Radboud University Medical Center Nijmegen Netherlands; 2 Radboud Center for Infectious Diseases Radboud University Medical Center Nijmegen Netherlands; 3 Department of Anesthesiology, Pain and Palliative Medicine Radboud University Medical Center Nijmegen Netherlands; 4 Department of Cardiac Surgery Radboud University Medical Center Nijmegen Netherlands

**Keywords:** EA-230, inflammation, pregnancy, cardiac surgery, immunomodulation, kidney/therapy, clinical trials, phase II as topic, safety

## Abstract

**Background:**

The cardiac surgery–induced systemic inflammatory response may induce postoperative hemodynamic instability and impairment of renal function. EA-230, a linear tetrapeptide (A-Q-G-V), is derived from the beta chain of the human chorionic gonadotropin pregnancy hormone. It has shown immunomodulatory and renoprotective effects in several animal models of systemic inflammation. In phase 1 and phase 2a studies, these immunomodulatory effects were confirmed during human experimental endotoxemia, and EA-230 was found to have an excellent safety profile.

**Objective:**

The objective of this first in-patient study is to test the safety and tolerability as well as the immunomodulatory and renoprotective effects of EA-230 in a proof-of-principle design in patients with systemic inflammation following on-pump cardiac surgery.

**Methods:**

We describe a prospective, randomized, double-blind, placebo-controlled study in which 180 elective patients undergoing on-pump coronary artery bypass grafting, with or without concomitant valve surgery, are enrolled. Patients will be randomized in a 1:1 ratio and will receive either EA-230 (90 mg/kg/hour) or a placebo. These will be infused at the start of the surgical procedure until the end of the use of the cardiopulmonary bypass. The primary focus of this first-in-patient study will be on safety and tolerability of EA-230. The primary efficacy end point is the modulation of the inflammatory response by EA-230 quantified as the change in interleukin-6 plasma concentrations after surgery. The key secondary end point is the effect of EA-230 on renal function. The study will be conducted in 2 parts to enable an interim safety analysis by an independent data monitoring committee at a sample size of 60. An adaptive design is used to reassess statistical power halfway through the study.

**Results:**

This study has been approved by the independent competent authority and ethics committee and will be conducted in accordance with the ethical principles of the Declaration of Helsinki, guidelines of Good Clinical Practice, and European Directive 2001/20/CE regarding the conduct of clinical trials. Results of this study will be submitted for publication in a peer-reviewed scientific journal. Enrollment of this study commenced in July 2016, and results are expected at the end of 2018.

**Conclusions:**

This adaptive phase 2 clinical study is designed to test the safety and tolerability of EA-230 in patients undergoing cardiac surgery. In addition, efficacy end points focused on the effect of the systemic inflammatory response and renal function are investigated.

**Trial Registration:**

ClinicalTrials.gov NCT03145220; https://clinicaltrials.gov/ct2/show/NCT03145220 (Archived by WebCite at http://www.webcitation.org/74JPh8GNN)

**International Registered Report Identifier (IRRID):**

DERR1-10.2196/11441

## Introduction

### Background

The systemic inflammatory response syndrome is characterized by a dysregulated inflammatory reaction in response to conditions such as a severe infection, trauma, and major surgery [1,2]. Although activation of the immune system is essential, a too-pronounced systemic inflammatory response may result in failure of 1 or more organ systems and is associated with morbidity and mortality rates up to 30% [3,4]. Development of acute kidney injury (AKI) represents an early and common manifestation of inflammation-induced organ failure [5-7].

During cardiac surgery, multiple insults, including sternotomy, application of cardiopulmonary bypass (CPB), and aortic cross-clamping are well known to contribute to a systemic inflammatory response [8-11]. The extent of this response is directly associated with impaired patient outcome, as elevated postoperative levels of interleukin (IL)-6 correlate with adverse outcomes and mortality [5,12]. Furthermore, this inflammatory response is believed to play a central role in the pathogenesis of AKI following cardiac surgery [13,14]. In addition, renal impairment is, in turn, independently associated with adverse outcome and impaired patient survival [15,16].

### Immunomodulation

Immunomodulatory strategies until now have failed to demonstrate clear beneficial effects in cardiac surgery patients [17]. For example, large trials on the use of high-dose corticosteroids did not improve overall patient outcome in cardiac surgery patients [18,19], although positive effects on respiratory variables in selected patient groups may be present, as found in post hoc analyses [19]. Nonspecific anti-inflammatory effects and the broad spectrum of side effects of these interventions may have contributed to the lack of overall beneficial effects of these compounds. As a result, current strategies consist of supportive treatment, and novel strategies aimed to attenuate the exaggerated proinflammatory response remain highly warranted.

Of interest, pregnancy is associated with an immune-tolerant adaptation of the immune system, necessary to facilitate the symbiosis of 2 major histocompatibility complex incompatible individuals [20]. Likely related to this effect, a remarkable improvement of several immune-mediated inflammatory diseases is observed during pregnancy [21-23]. Nevertheless, pregnant women are eminently capable of combating infections and often produce antibodies against paternal alloantigens of the fetus, demonstrating that they are fully immunocompetent. These features are suggestive of a selective modulation of the immune system in such a way that harmful immune processes to mother and fetus are suppressed, whereas beneficial immune processes remain unaffected. In this context, an array of oligopeptides related to the primary structure of the human chorionic gonadotropin (hCG) pregnancy hormone was designed and evaluated in experimental animal models of systemic inflammation [24-29]. Of the evaluated oligopeptides, the linear tetrapeptide (sequence: A-Q-G-V), now named EA-230, was shown to exert immunomodulatory effects and to protect against organ failure and associated mortality [25,26,28,30,31]. In particular, the administration of EA-230 resulted in renal function preservation, for example, in ischemia-reperfusion and kidney transplant models [30,31]. It is probable that the profound effects of pregnancy on renal function through increasing renal flow and subsequently increasing glomerular filtration rate (GFR) are causal to these findings [32]. Phase 1 safety studies of EA-230 showed that intravenous administration is well tolerated and has an excellent safety profile [33]. In a phase 2a study during human experimental endotoxemia, a model of controlled systemic inflammation induced by the administration of endotoxin, no safety concerns emerged. Furthermore, subjects treated with the highest dose of EA-230 (90 mg/kg/hour) resulted in less flu-like symptoms, attenuated development of fever, and reduced levels of proinflammatory mediators (among others IL-6 and IL-8) when compared with placebo-treated endotoxemia subjects [34].

### Objectives

A proof-of-principle study is now warranted to (1) investigate the safety profile of EA-230 in patients, (2) investigate whether EA-230 is able to modulate the systemic inflammatory response in patients, and (3) explore whether this translates into a clinical benefit in terms of prevention of organ dysfunction, in particular, renal injury. In this paper, we describe the design of a double-blind, placebo-controlled, randomized, adaptive phase 2 study with EA-230 in patients undergoing elective on-pump cardiac surgery.

## Methods

### Design and Setting

This study is a single-center, prospective, double-blind, placebo-controlled, randomized, single-dose phase 2 study. It has an adaptive design to evaluate the safety and immunomodulatory effects of EA-230 in patients undergoing on-pump cardiac surgery for coronary artery bypass grafting (CABG) with or without concomitant valve surgery. A total of 180 eligible patients are planned for inclusion and will be randomized to receive either active or placebo treatment in a 1:1 ratio. The study will be conducted in a tertiary hospital, the Radboud University Medical Center, Nijmegen, the Netherlands. This is the first-in-patient safety and tolerability study, of which the primary efficacy objective is to assess the immunomodulatory effects of EA-230. The key secondary efficacy end point is the effect of EA-230 on renal function. With regard to safety in this first-in-patient study, the study will be conducted in 2 parts. In the first part, 60 patients (40-50 low-risk patients) will be included (see Textboxes 1 and 2 for details) followed by an independent safety analysis by the data monitoring committee (DMC). Patient enrollment will only continue if no safety concerns are raised, and more high-risk patients will be included.

In addition, an adaptive design is used to re-evaluate the statistical power and group size of the study using patient data obtained during the first half of the study. This study is described in accordance with the Standard Protocol Items: Recommendations for Interventional Trial guidelines [35] and registered at ClinicalTrials.gov (identifier: NCT03145220).

### Study Objectives

#### Primary Objectives

The primary objectives of the study are to (1) assess the safety and tolerability of EA-230 in patients undergoing on-pump cardiac surgery (related to safety) and (2) assess the immunomodulatory effects of EA-230 in patients with systemic inflammation following on-pump cardiac surgery (related to efficacy).

#### Key Secondary Objective

The key secondary objective is to assess the effects of EA-230 on changes in renal function (GFR).

All end points, including other explorative efficacy end points, are described in Table 1.

**Inclusion criteria.**

1. Coronary artery disease, scheduled for elective on-pump coronary artery bypass grafting surgery with or without concomitant valve surgery
2. Written informed consent to participate in this study before any study-mandated procedure
3. Patients aged above 18 years, both male and female
4. Patients have to agree to use a reliable form of contraception with their partners from study entry until 3 months after study drug administration

**Exclusion criteria.**

1. Immune compromised
  - Solid organ transplantation
  - Known HIV
  - Pregnancy
2. Use of immunosuppressive drugs (list provided in Web-based supplementary material; see Multimedia Appendix 1)
3. Nonelective/emergency surgery
4. Hematological disorders
  - Known disorders from myeloid and/or lymphoid origin
  - Leucopenia
5. Known hypersensitivity to any excipients of the drug formulations used
6. Treatment with investigational drugs or participation in any other intervention clinical study within 30 days before study drug administration
7. Inability to personally provide written informed consent (eg, for linguistic or mental reasons)
8. Known or suspected of not being able to comply with the study protocol

Additional exclusion criteria to select low-risk patients (for the first 60 patients only)

1. EuroSCORE II >4
2. Renal function impairment: serum creatinine >200 µmol/L
3. Liver function impairment: alanine aminotransferase/aspartate aminotransferase >3 times above upper level of reference range
4. Left ventricular dysfunction: ejection fraction <35%
5. Coronary artery bypass grafting procedure with concomitant valve surgery

**Table 1:** End points.

Categories and measures			Period
**Main category**			
	**Safety and tolerability**		
		(Serious) adverse events	Signing of informed consent form to day 90
		Vital signs (heart rate and blood pressure)	First 24 hours of intensive care unit (ICU) admission
		Laboratory parameters (hemoglobin, hematocrit, leukocytes, thrombocytes, leukocyte differential blood count, sodium, potassium, creatinine, urea, alkaline phosphatase, alanine aminotransferase, aspartate aminotransferase, gamma glutamyl transferase, creatine kinase, bilirubin, and C-reactive protein)	Day −1 to day +1
**Efficacy**		
	Primary: Effect of EA-230 on the inflammatory response quantified by the change in IL^a^-6 plasma concentration over time (AUC^b^).		Surgery day to day 1
	Key secondary: Effect of EA-230 on GFR^c^ quantified by plasma clearance of iohexol (iGFR^d^)		Day −1 to day +1
**Explorative category**			
	**Inflammatory**		
		Effect of EA-230 on the inflammatory response quantified by the change in plasma concentrations over time of IL-6, IL-8, IL-10, tumor necrosis factor-α, IL-1 receptor antagonist, monocyte chemoattractant protein-1, macrophage inflammatory protein 1 (MIP1)^e^-α, MIP1-β, vascular cell adhesion molecule, intercellular adhesion molecule, and IL-17a	Surgery day to day 1
		Effect of EA-230 on leukocyte kinetics quantified by change of total cell counts over time	Day −1 to day +1
		Effect of EA-230 on changes in body temperature in degree Celsius over time	First 24 hours of ICU admission
		Effect of EA-230 on required insulin infusion rates	First 24 hours of ICU admission
	**Renal**		
		Effect of EA-230 on GFR estimated by modification of diet in renal disease calculation	Day −1 and day +1
		Effect of EA-230 on GFR measured by endogenous creatinine clearance using urine and plasma creatinine	Surgery to day +1
		Effect of EA-230 on plasma creatinine and proenkephalin	Day −1 to day +1
		Effect of EA-230 on changes in urine output	Surgery to day +1
		Effect of EA-230 on changes in urinary renal damage markers over time of kidney injury marker-1, neutrophil gelatinase–associated lipocalin, L-fatty acid–binding protein, tissue inhibitor metalloproteinase-2 and insulin-like growth factor binding protein-7, urinary IL-18, and *N*-acetyl-β-D-glucosaminidase	Surgery to day +1
		Effect of EA-230 on changes in urea, sodium, creatinine, and albumin in urine over time	Surgery to day +1
		Modulation in need for and duration of renal replacement therapy	Surgery to day 90
		Modulation in incidence of different stages of AKI^f^ according to the RIFLE^g^ criteria	Surgery to day 90
	**Cardiovascular**		
		Modulation in vasopressor use expressed as inotropic score: (dopamine dose × 1 μg/kg/min) + (dobutamine dose × 1 μg/kg/min) + (adrenaline dose × 100 μg/kg/min) + (noradrenaline dose × 100 μg/kg/min) + (phenylephrine dose × 100 μg/kg/min) + (vasopressin (mUnits/kg/min) × 10,000) + (milrinone × 10 mcg/kg/min) [36]	First 24 hours of ICU admission
		Effect of EA-230 on use of fluid therapy and fluid balance	First 24 hours of ICU admission
		Effect of EA-230 on creatine kinase and troponine T plasma concentration	Surgery to end of hospital stay
		Effect of EA-230 on thorax drain production	Surgery to end of ICU admission
	Pulmonary: Effect of EA-230 on alveolar-arterial gradient O_2_ gradient		Surgery to end of ICU admission
	**General outcome**		
		Effect of EA-230 on change in SOFA^h^ score over time	First 24 hours of ICU admission
		Effect of EA-230 on APACHE^i^ IV score at ICU admission	Surgery to ICU admission
		Effect of EA-230 on major clinical adverse events within 90 days (stroke, myocardial infarction, rethoracotomy, hospital readmission, and pleural and/or pericardial puncture)	Signing of informed consent form to day 90
		Effect of EA-230 on length of stay on ICU	Surgery to day 90
		Effect of EA-230 on length of hospital stay	Surgery to day 90
		Effect of EA-230 on 28 and 90 days mortality	Surgery to day 90
	**Pharmacokinetics of EA-230**		
		Peak blood plasma levels of EA-230	10 min before start and stop of CPB^j^
		Blood plasma levels of EA-230, AUC, maximal concentration, terminal half-life, clearance, and volume of distribution for a limited number of patients receiving active medication (n=15)	During EA-230 administration to 6 hours after stoppage

^a^IL: interleukin.

^b^AUC: area under the curve.

^c^GFR: glomerular filtration rate.

^d^iGFR: glomerular filtration rate measured by plasma clearance of iohexol.

^e^MIP1: macrophage inflammatory protein.

^f^AKI: acute kidney injury.

^g^RIFLE: risk, injury, failure, loss of kidney function, and end-stage kidney disease classification.

^h^SOFA: sepsis-related organ failure assessment score.

^i^APACHE: acute physiology and chronic health evaluation.

^j^CPB: cardiopulmonary bypass.

### Patient Selection or Eligibility

All adult patients (aged >18 years) scheduled for elective on-pump CABG procedure with or without concomitant valve surgery will be screened for eligibility; see Textboxes 1 and 2 for an overview of all inclusion and exclusion criteria.

### Recruitment

**Figure 1:**
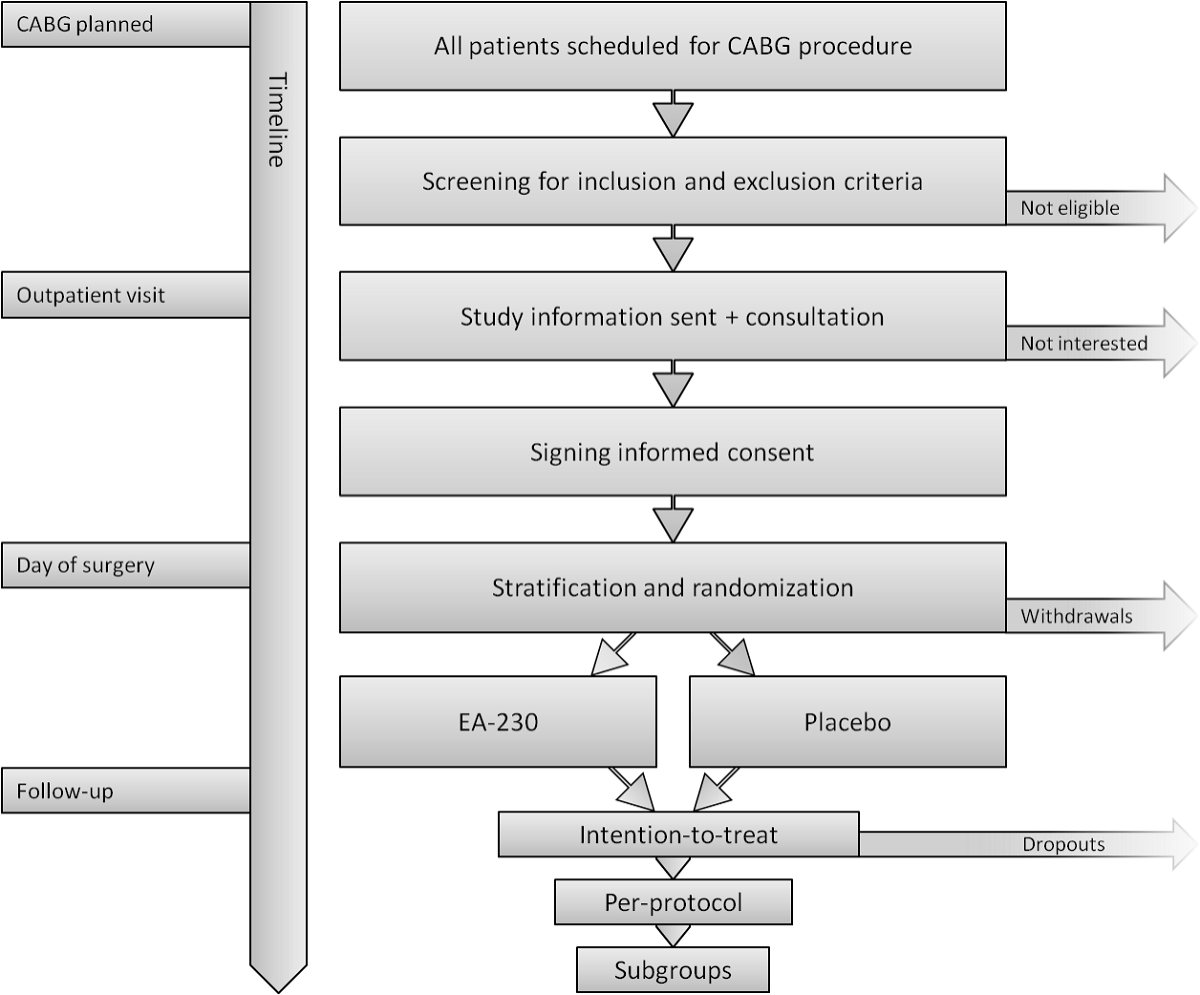
Study flowchart. Overview of patient recruitment, randomization, and population analysis procedures from screening to follow-up. CABG: coronary artery bypass grafting.

Figure 1 depicts a schematic flowchart of patient recruitment and randomization.

All patients scheduled for elective CABG surgery will be included in a screening log and informed through a detailed informative brochure. After screening for inclusion and exclusion criteria, eligible patients will be personally consulted, and a final inclusion and exclusion check will be performed. After obtaining written informed consent, patients will be enrolled into the study.

### Randomization and Stratification

On the morning of surgery, patients will be randomized by nonblinded independent study personnel for active or placebo treatment. Study personnel will use Good Clinical Practice– approved data management software (Castor EDC, Amsterdam, the Netherlands) in this process. The Castor system applies a stratified randomization to ensure equal distribution between active and placebo treatment of patients with known risk factors for adverse outcomes. Moreover, 3 strata will be included: (1) a CABG procedure with or without concomitant valve surgery; (2) preoperative renal function with an estimated GFR of ≤30, 31 to 90, and >90 mL/min/1.73 m^2^; and (3) a EuroSCORE II of <4 or ≥4 [37].

### Blinding

Double-blind conditions will be maintained for all patients, the attending physicians, and the medical study team personnel involved in all blinded study procedures, data collection, and/or data analyses. Nonblinded study personnel not involved in any other study procedures will prepare the study medication. Infusion systems and solutions for active and placebo treatment are identical in appearance and texture. The interim safety analysis will be performed by an independent data safety and monitoring board in an unblinded fashion. If it is decided that the study can be continued, all study personnel remain blinded. Unblinding will be authorized by the sponsor after completion of the study, performance of a blinded data review, and locking of the database. A sealed code break envelope is present in case emergency unblinding should be necessary.

### Study Intervention

Intravenous infusion of EA-230, 90 mg/kg/hour, or placebo, will be initiated at the moment of the first surgical incision using an automated infusion pump. Infusion rate is 250 mL/hour, and infusion will be continued until cessation of the CPB or after 4 hours of continuous infusion, whichever comes first.

EA-230 formulation is packed in sterile 5-mL glass vials, containing 1500 mg/vial, dissolved in water for injection at a final concentration of 300 mg/mL with an osmolality of 800 to 1000 mOsm/kg. The placebo formulation consists of sodium chloride diluted in water for injection in identical sterile 5-mL glass vials containing 29 mg/mL to reach a solution with an identical osmolality. EA-230 and placebo will be prepared for continuous intravenous infusion with an osmolality of <400 mOsm/kg by adding the appropriate amount of EA-230 or placebo to 1000 mL normal saline under aseptic conditions. Placebo and active treatment vials, manufactured by HALIX BV (Leiden, the Netherlands), will be provided by the sponsor.

### Outcome Measures

**Figure 2:**
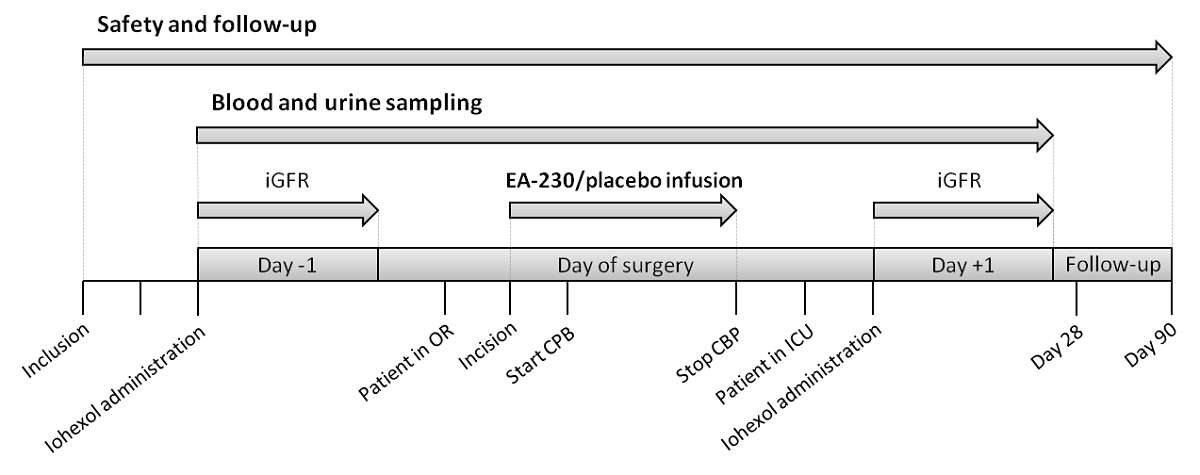
Timeline of study procedures. CPB: cardiopulmonary bypass; ICU: intensive care unit; iGFR: glomerular filtration rate measured by plasma clearance of iohexol; OR: Operating Room.

An overview of the study procedures from inclusion criteria until end of follow-up is depicted in Figure 2. A detailed overview of all outcome measures is also provided in Table 1.

#### Primary End Point

The primary focus is the safety and tolerability of EA-230. This is defined by the combination of several safety measurements: the incidence and severity of serious adverse events (SAEs) and serious unexpected suspected adverse reactions (SUSARs), the course of vital signs (heart rate and blood pressure), and the course of routine laboratory parameters. Vital signs and routine laboratory parameters will be registered during the first postoperative day when patients are admitted to the intensive care unit. Safety data will be collected from inclusion in the study until 90 days after the administration of the study drug.

The primary efficacy end point is the modulation of the inflammatory response by EA-230. This will be quantified by the difference in the area under the curve (AUC) plasma IL-6 levels over time from the start of the cardiac procedure until the first postoperative day compared with placebo. Plasma samples will be collected preincision (baseline) at the start of CPB; at 0, 2, 4, and 6 hours after cessation of the CPB; and on the morning of the first postoperative day.

#### Key Secondary End Point

Modulation of changes in renal function by EA-230 is defined by changes in the GFR measured before surgery and on the morning of the first postoperative day. For the determination of the GFR, an intravenous bolus of 5 mL iohexol will be administered, and plasma samples will be collected in the following 4 hours. On the day before surgery: 90 and 240 min after iohexol administration and on the postoperative day: 90, 180, and 240 min after iohexol administration. A plasma disappearance curve of iohexol will be constructed to calculate the iohexol GFR (iGFR) according to the methods described by Delanaye et al [38].

All other secondary efficacy end points are exploratory. Data for both parts of the study will be combined to assess both the safety and efficacy of the primary and secondary end points.

### Sample Size Calculation

The primary efficacy end point, AUC of plasma IL-6 levels over time, was used for the power calculation. In the preceding clinical phase 2a study with EA-230, during experimental endotoxemia in healthy volunteers, EA-230 (90 mg/kg/hour) attenuated AUC plasma IL-6 levels by 48% compared with placebo. This first-in-patient proof-of-principle study is powered on a 30% reduction in AUC IL-6, which is deemed a relevant immunomodulatory effect. For the statistical dispersion, AUC IL-6 data from a previous CABG surgery study conducted in the same institute were used (mean 816 pg/mL/hour, SD of 520 pg/mL/hour) [39]. To correct for the nonparametric distribution of these data, the calculated sample size is increased by 15% [40]. With a 2-sided alpha of .05 and a power of 80% (beta of .2), a group size of 82 patients per treatment arm is required. However, selection of low-risk patients in the first part of the study with an expected less pronounced inflammatory response may result in an increased SD of AUC IL-6 in the overall study, and therefore, loss of study power. Hence, the sample size should be adjusted accordingly. In consultation with an independent statistician, a sample size of 90 patients per treatment arm was deemed sufficient to compensate for this loss in power. Using an adaptive design, sample size will be re-evaluated, and possible early efficacy will be assessed halfway through the study, when 90 patients have been included. A partially unblinded independent statistician and member of the DMC will perform these analyses. For the re-evaluation of the sample size, the pooled SD of the AUC plasma IL-6 levels of the first 90 patients enrolled will be calculated and used to compare with the original sample size according to the method described by Proschan [41]. To demonstrate possible early efficacy, the approach for alpha-spending according to O’Brien-Fleming will be used [42]; a *t* test will be performed on the collected data with the following alpha (α1(t*)):α1(t*)=2−2φ(Z α/2/√t*) where t* represents the information fraction (t*=0.5 × original sample size/new sample size). If *P*<α1(t*), the study will be stopped because of early demonstrated efficacy. When no significant differences are found during this interim analysis, the study will continue. For final analysis, an adjusted alpha will be used, corrected for alpha spending.

### Statistical Analysis

The safety parameters will be listed and summarized descriptively according to treatment. No statistical testing on safety end points will be performed. The statistical analysis plan for efficacy end points will be signed before database lock and is provided as online supplementary material (see Multimedia Appendix 2). Data will be presented as mean and SD or SE of the mean or median and interquartile range and analyses performed, depending on their distribution. The primary efficacy end point, the difference in IL-6 plasma concentrations over time (AUC IL-6 plasma levels) between treatment groups, will be analyzed using an unpaired Student *t* test or Mann-Whitney U test (the latter if data are not normally distributed). In a secondary analysis, the AUC IL-6 plasma levels between treatment groups will also be compared using 2-way analysis of variance (ANOVA; interaction term, on log-transformed data if data are not normally distributed). Differences in the key secondary efficacy end point iGFR between treatment groups over time will be analyzed using 2-way ANOVA, as described above. All other data will be analyzed using unpaired Student *t* tests or Mann-Whitney U tests for continuous data, 2-way ANOVA for continuous data over time as described above, and chi-square tests for categorical data. A 2-sided *P* value <.05 is considered significant. For the primary end point, a *P* value corrected for alpha spending will be used as described earlier. Statistical analyses will be performed using IBM SPSS (IBM, Armonk, NY, USA) and GraphPad Prism (GraphPad Software, La Jolla, CA, USA).

### Withdrawal of Study Patients

Patients may leave the study at any time, for any reason, and without any consequences. The investigator can decide to withdraw a patient from the study for urgent medical reasons or in case of inability to comply with the study protocol.

There is a likely possibility that patients enrolled in the study have their cardiac surgery rescheduled as a result of urgent intervening surgeries or because they meet an exclusion criterion shortly before the start of surgery. Therefore, patients who are withdrawn from the study before investigational medicinal product administration will be replaced and thus will not be included in any analysis.

### Different Populations to be Analyzed

#### Intention-to-Treat Population

The intention-to-treat (ITT) population includes all patients who were randomized and received study treatment, irrespective of satisfying other end point criteria. This population will be used for the analysis of safety and tolerability and all other primary and secondary end points.

#### Per-Protocol Population

Analysis of the per-protocol (PP) population will be used as a supplement to the ITT analysis and will be performed for all end points except safety-related end points. The PP includes all ITT patients who have not been excluded from analysis for major protocol deviations.

#### Pharmacokinetic Population

Sampling for pharmacokinetic (PK) population analysis will be performed in 30 patients. As EA-230/placebo ratio is 1:1, the PK population will include a subset of approximately 15 patients who received EA-230. For this full PK evaluation, additional blood samples will be obtained during infusion of EA-230 until 6 hours after cessation of administration.

#### Subgroup Analyses

Subgroup analyses will be performed on the following predetermined preoperative randomization strata: (1) CABG with or without concomitant valve surgery; (2) preoperative renal function with an estimated GFR of ≤30, 31 to 90, and >90 mL/min/1.73 m^2^; and (3) EuroSCORE II of <4 or ≥4.

### Safety Considerations

#### Adverse Events

All adverse events will be judged by the investigators with regard to severity (*mild, moderate, or severe*) according to Common Terminology Criteria for Adverse Events guidelines 4.030 [43] and their perceived relation to the study drug (*definitely, probably, possibly, or unrelated/unlikely to be related*). SAEs or SUSARs include death, life-threatening disease, persistent and/or significant disability and/or incapacity, and hospitalization and/or prolongation of inpatient hospitalization.

The investigator will report all SAEs and SUSARs to the sponsor without undue delay after obtaining knowledge of the events. Monitoring and re-evaluation of the SAE/SUSAR and its relation to the study drug will subsequently be performed by an independent medical doctor with the independent Contract Research Organization QPS (Groningen, the Netherlands) and reported to the Central Committee on Research Involving Human Subjects (CCMO, The Hague, The Netherlands) within a maximum period of 15 days (or 7 days when the event is life threatening or results in death) and to the independent ethics committee of the Radboud University Nijmegen Medical Center (CMO, Arnhem-Nijmegen, the Netherlands).

#### Data Monitoring Committee

An independent DMC, consisting of 3 expert members, including 1 biostatistician, will assess safety of the study drug. The first DMC meeting will be held on completion of the first part of the study that includes 40 to 50 low-risk patients. During this interim safety analysis, inclusion will be paused, and an extensive partially unblinded assessment of (S)AEs, vital signs, and routine laboratory parameters will be performed. Inclusion of patients in the study will only continue if no safety concerns are raised by the DMC. A second meeting will be held after 90 patients have been enrolled (including higher risk patients) to reassess all safety parameters. At this point, the DMC will advise on the continuation or termination of the study.

### Ethical Considerations

The regional and central independent ethical committee, CMO Arnhem-Nijmegen, and the CCMO, respectively, have approved the study protocol, amendments, informed consent, and all other study-relevant written information for the patients. The study will be conducted in accordance with the ethical principles of the Declaration of Helsinki ICH E6(R1), the Medical Research Involving Human Subjects Act, guidelines of Good Clinical Practice, and European Directive (2001/20/CE). Informed consent will be obtained before any study-specific procedures are performed. Substantial amendments will be provided to the CMO for approval. Nonsubstantial amendments will be provided to the CMO for notification.

### Data Quality Assurance and Publication

Data will be handled confidentially and anonymously. The study site maintains source documentation and is responsible for accurate data entry in electronic case report form. The Good Clinical Practice–certified data capture system Open Clinica (Waltham, MA, USA) was used in this process. Blinded study personnel are provided with an individual username and password with complete traceability. Quality assurance, data management with full data validation and monitoring of all source documents, study procedures, study data, SAEs, and SUSARs will be performed by the independent Contract Research Organization QPS. The database will be locked after completion of the data review, resolutions to all queries, and the signing of the statistical analysis plan. Following database lock, a study patient identification code list (provided by the Castor data management system, Amsterdam, the Netherlands) will be used to link the stratified interventional treatment (active or placebo) to the patient. Data and body material will be kept in secure storage at the intensive care research department and is accessible by study personnel only. The handling of patient data in this study complies with the Dutch Personal Data Protection Act (in Dutch: Wet Bescherming Persoonsgegevens). The principal investigator and the subinvestigators will write the manuscript that will be submitted for publication in a peer-reviewed scientific medical journal after completion of the study, irrespective of results.

### Patient and Public Involvement

Patients and the public were not involved in the design and/or the conduct of the study protocol. Study outcome will be disseminated to all study participants individually. The burden of the intervention was assessed by the independent ethics committees CMO and CCMO, which include laymen members.

## Results

Enrollment of this study commenced in July 2016, and results are expected at the end of 2018.

## Discussion

### General

To evaluate the safety, immunomodulatory, and renoprotective effects of EA-230, we designed this single-center, double-blind, randomized, placebo-controlled adaptive phase 2 study in patients undergoing on-pump cardiac surgery. In this study primarily focused on safety, we will also assess the immunomodulatory effects of EA-230. Our key secondary objective is to investigate whether EA-230 prevents a decrease in renal function (which is possibly related to its immunomodulatory effects) in this patient population. Patients undergoing on-pump cardiac surgery represent an ideal study population to evaluate the effects of EA-230 on the systemic inflammatory response in a proof-of-principle study. These cardiac surgery procedures are highly standardized and have a well-characterized sequence of inflammatory insults: release of Danger-Associated Molecular Pathways as a result of tissue damage during incision and sternotomy, leukocyte activation induced by the use of the extracorporeal circuit, ischemia- reperfusion damage following aortic cross-clamping and subsequent declamping, and translocation of endotoxins because of increased gut permeability [8-11]. Due to the elective timing of these insults, the moment of activation of the immune system is well defined, and the following inflammatory response during and after surgery follows a relatively homogenous pattern. Furthermore, the inflammatory response is clinically relevant, as inflammatory mediators IL-6 and IL-8 have been shown to be key orchestrators of the systemic inflammatory response following cardiac surgery and are associated with postoperative adverse outcome, including the occurrence of AKI and long-term mortality [5,12,44]. As EA-230 has attenuated IL-6 and IL-8 in earlier work [34], it has the potential to modulate release of these specific mediators that are associated with organ failure in cardiac surgery patients.

### Design

The inclusion and exclusion criteria of this study are not overly restrictive to facilitate enrollment and to increase generalizability. Although the inflammatory response is relatively comparable among cardiac surgery patients, these permissive criteria might influence study results because patients with known preoperative risk factors may develop a more pronounced inflammatory response and/or have a worse outcome. However, in a randomized study such as this, these interpatient differences are likely to be equally divided between treatment and placebo groups. Nevertheless, we used a stratified randomization procedure to optimize group balance. In addition, 3 major preoperative determinants for outcome, namely baseline renal function, EuroSCORE II [37], and CABG with or without valve surgery were used to ensure equal distribution between groups of patients with known risk factors.

The 2-part study design facilitates an extensive safety interim analysis by the DMC after the first part and limits potential risks for patients. It also ensures efficient assessment of efficacy by combining data from both parts for all safety and efficacy analyses. Furthermore, with the use of an adaptive design, the trial sample size can be adjusted halfway through the trial by conducting a new power calculation using the variation of IL-6 concentrations of obtained data of the first half of the study, this way guaranteeing adequate statistical power. The use of such an adaptive design has recently been recommended for the design of clinical studies such as this study [45].

To date, no side effects attributed to EA-230 have been observed, which is in sharp contrast to other immunomodulatory drugs. This may be because of the fact that EA-230 is an endogenous, immunological active breakdown product of the pregnancy hormone beta hCG. In addition, the fact that the tolerant immune phenotype during pregnancy is not accompanied with complications related to immunosuppression suggests that targeting this pathway may be of more benefit than the use of other immunomodulatory therapies.

Evaluation of the effects of EA-230 on renal function is of specific interest in this study for several reasons. First, EA-230 exerted potent renal protective effects in animal studies. Second, a significant proportion of these patients suffer from postoperative renal injury [13,14], which is, in turn, related to their clinical outcome [15,16]. This study is unique in terms of accurate assessment of renal function because a gold standard method to measure GFR is used, instead of estimating GFR based on serum creatinine. Although it is well established that serum creatinine and urine output are suboptimal parameters to assess acute deterioration of renal function, they remain to be the most commonly used markers to diagnose AKI clinically and in research settings. Creatinine is unreliable because it is a late marker and is influenced by muscle mass, fluid shifts, immobilization and is partially, but actively, secreted by the kidneys [46-48]. The iGFR method has proven to be as reliable as the inulin clearance (with an *R*^*2*^ of 0.96 [49]) and accurately detects even minor changes [38,50]. Therefore, clinically significant changes in GFR can be reliably assessed in this study. These renal function measurements have not been performed in any large cardiac surgery trial to date and will substantially improve the validity of the data and quality of this study. As a potential downside, the iohexol method does require trained personnel in the collection of multiple blood samples to create a plasma decay curve. As a result of this labor-intensive process, iGFR measurements will be performed only twice in this study: preoperatively, representing a baseline measurement and on the morning of the first postoperative day. As discussed earlier, conventional renal function markers are late and unreliable. Therefore, little is known about the exact postoperative course of renal function deterioration and/or decrease in GFR. As such, there is a chance that the single postoperative iGFR measurement could fail to detect a decrease in GFR following on-pump cardiac surgery.

A limitation of this study is the dose of EA-230. Only one dose is used, based on the fact that only the highest dose was effective in terms of modulating the immune response in the previous experimental human endotoxemia study [34]. As a result, it will remain unknown whether similar efficacy in patients can be attained using a lower dose or higher efficacy can be achieved using a higher dose. Furthermore, whether the use of the artificial extracorporeal circulation affects EA-230’s pharmacokinetics or pharmacodynamics is unknown. Along these lines, the fluid balance shift in patients undergoing cardiac surgery may alter distribution of EA-230, with nontherapeutic plasma concentrations as a possible result.

### Summary

The EASI Study is a double-blind, randomized, placebo- controlled phase 2 study of EA-230 in 180 patients undergoing on-pump cardiac surgery. It applies stratification and has an adaptive study design. Apart from safety and tolerability, it is designed to examine the immunomodulatory and renal protective effects of EA-230 in patients with systemic inflammation.

## Appendix

Multimedia Appendix 1 List of immunosuppressive drugs.

Multimedia Appendix 2 Statistical Analysis Plan.

## Abbreviations

AKI: acute kidney injury
ANOVA: analysis of variance
AUC: area under the curve
CABG: coronary artery bypass grafting
CCMO: Central Committee on Research Involving Human Subjects
CMO: independent ethics committee of the Radboud University Nijmegen Medical Center
CPB: cardiopulmonary bypass
DMC: data monitoring committee
GFR: glomerular filtration rate
hCG: human chorionic gonadotropin
ICU: intensive care unit
iGFR: glomerular filtration rate measured by plasma clearance of iohexol
IL: interleukin
ITT: intention-to-treat
MIP1: macrophage inflammatory protein
PK: pharmacokinetic
PP: per-protocol
RIFLE: risk, injury, failure, loss of kidney function, and end-stage kidney disease classification
SAE: serious adverse event
SOFA: sepsis-related organ failure assessment score
SUSAR: serious unexpected suspected adverse reaction
