# Supplementary material for: The Safety, Tolerability, and Effects on the Systemic Inflammatory Response and Renal Function of the Human Chorionic Gonadotropin Hormone-Derivative EA-230 Following On-Pump Cardiac Surgery (The EASI Study): Protocol for a Randomized, Double-Blind, Placebo-Controlled Phase 2 Study
Source: JMIR Res Protoc. 2019 Feb 6;8(2):e11441. doi: 10.2196/11441 (PMC6381408; doi:10.2196/11441)
Supplement: Multimedia Appendix 1 [file resprot_v8i2e11441_app1.pdf]

**Immunosuppressive agent**	**Upper limit dosage use**
Corticosteroid<br>Prednisone<br>Hydrocortisone<br>Methylprednisolone<br>Dexamethasone<br>Cortisone<br>Betamethasone	>10 mg/day of prednisone or its equivalent daily<br>10 mg<br>40 mg<br>8 mg<br>1.5 mg<br>50 mg<br>1.2 mg
Methotrexate (Rheumatrex, Trexall)	Excluded at any dose
Leflunomide (Arava)/ Teriflunomide (Aubagio)	Acceptable if being used as monotherapy
Cyclophosphamide (Cytoxan)	Excluded at any dose
Cyclosporine A	Excluded at any dose. Ophthalmic formulation (Restatis) is permitted.
FK 506 (Tacrolimus)	Excluded at any dose. Topical formulation (Protopic) is permitted.
Azathioprine	Excluded at any dose
Cancer chemotherapy	Patients having received cancer chemotherapy in the previous 4 weeks are excluded.
Mycophenolate Mofetil, MMF (CellCept)	Solid organ transplant and bone marrow transplant patients are excluded.
Sirolimus (Rapamycin, rapamune)	Solid organ transplant and bone marrow transplant patients are excluded.
Everolimus (Certican)	Excluded at any dose.
Temisirolimus (Torisel)	Excluded at any dose.
Thalidomide	Patients receiving this drug within the past 72 hours are excluded
**Biologics**	
Anti-tumor necrosis factor (TNF) agents<br>Etanercept (Enbrel), Adalimumab (Humira), Infliximab (Remicade), Certolizumab (Cimzia), Golimumab (Simponi)	Patients receiving anti-TNF agents within the past 8 weeks are excluded.
Interleukin-1 Receptor antagonist (IL-1 RA) (Kineret)	Patients receiving IL-1 RA within the past 8 weeks are excluded
CTLA-4 Fusion protein	Patients receiving CTLA-4 Fusion protein within the

Atapacept (Orencia)<br>Belatacept (Nulojix)	past 8 weeks are excluded.
Anti-C20<br>Rituximab (Rituxan/MabThera)<br>Obintuzumab (Gazyva)	Patients receiving this drug within the past 2 years are excluded.
Anti-CD52<br>Alemtuzumab (Campath)	Patients receiving this drug within the past 2 years are excluded.
Anti-IL2<br>Daclizumab or Anti-Tac (Zenapax)<br>Basiliximab (Simulect)	Patients receiving any of the drugs within the past 2 years are excluded.
Anti-IL6<br>Tocilizumab (Actemra/RoActemra)	Patients receiving this drug within the past 2 years are excluded.
Anti-IL12/13<br>Ustekinumab (Stelara)	Patients receiving this drug within the past 2 years are excluded
Anti-BAFF (B-cell activating factor)<br>Belimumab (Benlysta)	Patients receiving this drug within the past 2 years are excluded.
Integrin inhibitor<br>Natalimumab (Tysarbi)	Patients receiving this drug within the past 2 years are excluded.
