# Supplementary material for: The Safety, Tolerability, and Effects on the Systemic Inflammatory Response and Renal Function of the Human Chorionic Gonadotropin Hormone-Derivative EA-230 Following On-Pump Cardiac Surgery (The EASI Study): Protocol for a Randomized, Double-Blind, Placebo-Controlled Phase 2 Study
Source: JMIR Res Protoc. 2019 Feb 6;8(2):e11441. doi: 10.2196/11441 (PMC6381408; doi:10.2196/11441)
Supplement: Multimedia Appendix 2 [file resprot_v8i2e11441_app2.pdf]

Radboudumc Intensive Care
QPS helps you navigate

# STATISTICAL ANALYSIS PLAN

| | |
|---|---|
| Protocol Title: | Randomized double blind placebo-controlled phase II study on the effects of **EA**-230 on the **S**ystemic **I**nflammatory response following on-pump cardiac surgery. The **EASI**-study. |
| Sponsor's Protocol Number: | EBI-EA230-CABG-2015 |
| QPS code | 150159 |
| Sponsor Name and Address: | Exponential Biotherapies Inc.<br>Kneuterdijk 2, 2514 EN, The Hague, The Netherlands |
| Date and Version of SAP: | 25 May 2018, Final version 1 |
| Prepared by: | Remi Beunders, Roger van Groenendael, Peter Pickkers<br><br>Thembile Mzolo/ Denise Montagne/ Julie Yeh<br>Biostatistician<br><br>Thomas Chou, Ph.D.<br>Senior Principal Clinical Pharmacokineticist |

Confidentiality Statement
This Statistical Analysis Plan is the confidential information of QPS LLC and is intended solely for the guidance of the clinical investigation. This Statistical Analysis Plan may not be disclosed to parties not associated with the clinical investigation or used for any purpose without the prior written consent of QPS LLC.

Radboudumc
Intensive Care
QPS
helps you navigate

## QPS AUTHORS' SIGNATURE PAGE

**PREPARED BY:**

[signature] May 25, 2018

Julie Yeh — Date
Biostatistician, QPS-Qualitix

[signature] May 25, 2018

Thomas Chou, Ph.D. — Date
Senior Principal Clinical Pharmacokineticist, QPS, LLC

**REVIEWED BY:**

[signature] May 25, 2018

Samantha Su — Date
Supervisor of Biostatistics, QPS-Qualitix

**APPROVED BY:**

Peter Pickkers, MD, PhD — Date
Principal investigator, Radboud University Medical Centre

Roger van Groenendael, MD — Date
Coordinating investigator, Radboud University Medical Centre

Remi Beunders, MD — Date
Coordinating investigator, Radboud University Medical Centre

Radboudumc
Intensive Care
QPS helps you navigate

## QPS AUTHORS' SIGNATURE PAGE

### PREPARED BY:

Julie Yeh — Date
Biostatistician, QPS-Qualitix

Thomas Chou, Ph.D. — Date
Senior Principal Clinical Pharmacokineticist, QPS, LLC

### REVIEWED BY:

Samantha Su — Date
Supervisor of Biostatistics, QPS-Qualitix

### APPROVED BY:

Peter Pickkers, MD, PhD — Date: May 26th 2018
Principal investigator, Radboud University Medical Centre

Roger van Groenendael, MD — Date: May 26th 2018
Coordinating investigator, Radboud University Medical Centre

Remi Beunders, MD — Date: May 26th 2018
Coordinating investigator, Radboud University Medical Centre

**TABLE OF CONTENTS**

## Inhoud

1. LIST OF ABBREVIATIONS AND DEFINITIONS OF TERMS ..... 5
2. DOCUMENTS USED ..... 7
3. STUDY OBJECTIVES ..... 7
3.1 Objectives ..... 7
3.2 Data Sources ..... 7
4. OVERALL STUDY DESIGN AND PLAN ..... 7
4.1 Study Design ..... 7
4.2 Randomization ..... 8
4.3 Study Medication ..... 8
4.4 Sample Size ..... 8
4.5 Study endpoints ..... 9
4.6 Study Schedule ..... 10
Tabel 1 Study Schedule ..... 11
5. DESCRIPTION OF INCLUDED PATIENTS ..... 12
5.1 Analysis Populations ..... 12
5.1.1 Intent-to treat (ITT) Population ..... 12
5.1.2 Pharmacokinetic (PK) Population ..... 12
5.1.3 Per-Protocol (PP) Population ..... 12
5.1.4 Strata Populations / Subgroup Populations ..... 12
5.2 Status of the patients ..... 12
5.3 Protocol Deviations ..... 13
5.4 Creation and Documentation of the PP population ..... 13
6. STATISTICAL ANALYSES AND REPORT SPECIFICATIONS ..... 13
6.1 General Considerations ..... 13
6.2 Demographic and Baseline Characteristics ..... 14
6.3 Study Patients ..... 14
6.4 Analysis of Safety and Tolerability ..... 15
6.4.1 General Considerations ..... 15
6.4.2 Safety and Tolerability Variables ..... 15
6.4.3 Adverse Events ..... 15

6.4.4 Clinical Laboratory Evaluations .... 16
6.4.5 Vital Signs .... 17
6.5 Analysis of Efficacy Data .... 17
6.5.1 Primary Endpoint .... 17
6.5.2 Key Secondary Efficacy Endpoint .... 18
6.6 Analyses of Pharmacokinetics of EA-230 .... 20
6.6.1 PK Data Handling Procedures .... 21
7. CHANGES FROM PROTOCOL AND OTHER REMARKS .... 22
8. SOFTWARE .... 23
8.1 Coding Systems .... 23
8.2 Statistical Software .... 23
8.3 Reporting .... 23
9. TABLES, LISTINGS AND FIGURES .... 23
9.1 List of Tables and Figures .... 25
9.2 List of Patient Data Listings .... 28
Appendix A TEMPLATES OF TABLES, LISTINGS AND FIGURES ... **Fout! Bladwijzer niet gedefinieerd.**

## 1. LIST OF ABBREVIATIONS AND DEFINITIONS OF TERMS

| | |
|---|---|
| AE | Adverse Event |
| AUC | Area under the curve |
| AUC0-last | Area under the concentration-time curve calculated using linear up/log down trapezoidal summation from time zero to time t, where t is the time of the last concentration above the limit of quantification |
| AUC0-inf | Area under the concentration-time curve from time zero to infinity, AUC0-inf = AUC0-last + Ct/kel, where kel is the terminal elimination rate constant and Ct is the last concentration |
| %AUCextra | Percentage of extrapolated AUC obtained by Ct/kel compared to AUC0-inf |
| AUEC | Area under the effect–time curve according to the linear trapezoidal method using IL-6 concentration-time values from time zero to time t, where t is the time of the last IL-6 concentration (Ct) |
| BMI | Body Mass Index |
| BQL | Below the quantification limit |
| CABG | Coronary artery bypass grafting |
| Cl | Clearance |
| Cli | Iohexol Clearance |
| Cmax | Maximum observed concentration |
| CR | Clinically relevant |
| CRO | Contract Research Organization |
| CS | Clinically Significant |
| CSR | Clinical Study Report |
| CV% | Percentage coefficient of variation |
| DBL | Database lock |
| DSMB | Data Safety monitoring Board |
| ECG | Electrocardiogram |
| EEG | Electroencephalography |
| eCRF | electronic Case Report Form |
| GFR | Glomerular filtration rate |
| GM | Geometric mean |
| HR | Heart rate |
| ICF | informed consent form |
| ITT | Intent-to-Treat |
| kel | Apparent terminal elimination rate constant calculated by linear regression of the terminal linear portion of the log concentration vs. time curve |
| LCL | Lower confidence limit |
| LOQ | Limit of quantification |
| max | Maximum |
| MedDRA | Medical Dictionary for Regulatory Activity |
| MH | Medical History |
| mg | Milligram |
| min | Minimum |
| N, n | Population size (N for sample size, n for available data) |
| NCR | Not clinically relevant |
| NCS | Not clinically significant |
| PI | Principal Investigator |

| | |
|---|---|
| PP | Per-protocol population |
| PD | Pharmacodynamics |
| PK | Pharmacokinetic(s) |
| PK/PD AP | PK/PD Analysis Plan |
| PT | Preferred term |
| PT | Prothrombin time |
| PTT | Partial thromboplastin time |
| QC | Quality Control |
| RM | Repeated Measures |
| SAE | Serious AE |
| SAP | Statistical Analysis Plan |
| SD | Standard Deviation |
| SEM | Standard error of the mean |
| SIRS | Systemic inflammatory response |
| SOC | System Organ Classification |
| SRT | Safety Review Team |
| SUSAR | Suspected unexpected serious adverse reaction |
| TEAE | Treatment Emergent Adverse Event |
| Tmax | Time of the maximum concentration |
| t1/2 | Apparent terminal elimination half-life calculated as ln(2)/kel |
| UCL | Upper confidence limit |
| VS | Vital signs |
| Vz | Volume of distribution based on the terminal phase |
| WHO | World Health Organization |

## 2. DOCUMENTS USED

Study Protocol: EBI-EA230-CABG-2015, Version 4.5, April 2018

Source/CRF: eCRF, Version update 2, 22 March 2018

## 3. STUDY OBJECTIVES

### 3.1 Objectives

- To assess the safety and tolerability of EA-230 in patients undergoing cardiac surgery with cardiopulmonary bypass.
- To assess the immunomodulating effects of EA-230 in patients with systemic inflammation following cardiac surgery with cardiopulmonary bypass.
- To assess the putative renal protective effects of EA-230 in patients with systemic inflammation following cardiac surgery with cardiopulmonary bypass.
- To assess the effects of EA-230 on other clinical outcomes for these patients.
- To assess the pharmacokinetics of EA-230 in these patients with systemic inflammation

### 3.2 Data Sources

Analyses will be carried out using data from the eCRF (OpenClinica). In addition, vital signs, cytokines (including IL-6), insulin sensitivity, pharmacokinetics, renal function (using endogenous creatinine clearance and iohexol plasma clearance as a measure of GFR), APACHE IV and EuroSCORE data will be received in Excel format. These will be used for all statistical analyses.
Samples for PK analyses will be sent to QPS Delaware for further processing. PD statistical analyses will be performed by BS group in Taiwan.

## 4. OVERALL STUDY DESIGN AND PLAN

### 4.1 Study Design

This is a combined phase IIa and phase IIb trial with a prospective, monocentric, double-blind, placebo-controlled, randomized, single-dose adaptive-group size design in patients undergoing elective coronary artery bypass grafting (CABG) surgery with or without valve replacement. Patients randomized to active treatment will receive a single dose (via continuous infusion EA-230 of 90 mg/kg/hour during the surgical procedure), administered i.v. with an automatic infusion pump, with a maximum infusion duration of 4 hours. Patients will receive active (EA-230) or placebo treatment in a randomized double blind fashion in a 1:1 proportion (active/placebo treatment). In part 1 (phase IIa), a total of 30 patients receiving active treatment will be included, and an additional 30 patients receiving placebo. In part 2 (phase IIb), 60 patients per treatment group will be included. After enrolment in the study, all 180 patients will be monitored for a follow-up time of 90 days.

After inclusion of 60 patients in the first study part, a report containing all the relevant safety data including (S)AE's and SUSARs will be provided to the DSMB and to the ethics committee (CMO). After

unblinded analysis of all safety data, the DSMB will advice whether to proceed with part 2 of the study, or not. The study team and sponsor and all personell involved in the conduct and monitoring of the study remain blinded. Additionally, the statistical power and group size will be re-evaluated by the DSMB statistician halfway the trial, after 90 patients have been included (details are provided in protocol chapter 16.4). Safety data will be re-evaluated at a second interim analysis after a total of 90 patients have been enrolled (including patients from both part 1 and part 2).

### 4.2 Randomization

Stratified randomization will be performed by using the GCP-qualified data management program, Castor EDC. Stratification will be used to guarantee equal distribution between active and placebo treatment of patients with known risk factors for possible adverse outcome, resulting in balanced groups. Three strata are: CABG surgery with or without valve replacement, pre-operative kidney function with an estimated GFR of ≤30, 31-90 and >90 ml/min/1.73 $m^2$, and EuroSCORE II of <4 or ≥4.

### 4.3 Study Medication

Part 1: 60 patients & part 2: 120 patients

- EA-230 treatment: EA-230 90 mg/kg/hour continuous i.v. infusion during surgery for a maximum of 4 hours after first incision.
- Placebo treatment: NaCl 0.9% continuous i.v. infusion during surgery for a maximum of 4 hours after first incision.

### 4.4 Sample Size

For part 1 of the study, the primary endpoint is safety. Therefore, no formal sample size calculation was performed for this part. We regard a group size of 30 patients per treatment arm an adequate representation to assess the safety and tolerability of EA-230 in our patient population.
Anticipating the study will be continued to part 2, a power calculation is performed using the AUC of plasma levels of IL-6, as a representative of the innate immune response, to demonstrate the immunomodulatory effects of EA-230.
In our previous study with healthy volunteers, we showed that EA-230 (90 mg/kg/hour) significantly attenuated the AUC of plasma IL-6 levels by 48% during experimental endotoxemia (AUC of plasma IL-6 from t=0 to t=8h), compared to placebo. For the inflammatory response following cardiac surgery, an ongoing inflammatory stimulus over a longer period of time is expected, however with a less pronounced increase in IL-6 compared to experimental endotoxemia. Therefore, we will power the present study on a clinically relevant attenuation of 30% in the AUC of plasma IL-6 levels.

For the statistical dispersion, we use data on the AUC of IL-6, measured in a previous CABG study within the same institute: Mean AUC of IL-6 production over time was 816 pg/ml with an SD of 520 pg/ml, although data were not normally distributed. To correct for the non-parametric distribution in the power calculation, we use a correction factor of +15% on the calculated sample size. With an α of 0.05 and a β of 0.8, a group size of 81 (rounded to 80) patients per treatment would be required.

However, instead of using a total of 220 patients (n=60 for phase IIa (safety) and n=160 for phase IIb (efficacy)), it would be more efficient to combine efficacy data (AUC of IL-6 ) from part 1 and part 2. This way, less patients are required for part 2. Taken into account that for part 1 only low-risk patients are enrolled with an expected less pronounced inflammatory response (and thus less

pronounced increase in IL-6 levels), this may result in some loss of power. If we enroll a total of 180 patients (n=60 for phase IIa and n=120 for phase IIb), the power of the study will be 83.5% (with an α of 0.05). We regard this group size with an increase of 3.5% in power acceptable to compensate for the loss of power in the patient population of part 1 (phase IIa), while minimizing the amount of patients that need to be exposed to the study drug.

An additional adaptive power analysis will be performed to re-evaluate group size and power halfway the conduct of the study, after 90 patients have been included. Details are provided in study protocol chapter 16.4.

## 4.5 Study endpoints

The endpoints in this study are divided into three categories: Safety and Tolerability, Efficacy, and Pharmacokinetics.

**Safety and Tolerability:**

- Safety and tolerability of EA-230;
    - Adverse events
    - Vital signs (effects on heart rate and blood pressure)
    - Laboratory parameters (Hb, Ht, Leucocytes, thrombocytes, Leucocyte differential blood count, sodium, potassium, creatinine, urea, alkaline phosphatase, ALT, AST, γGT, CK, bilirubin, CRP)

**Efficacy:**
**Primary efficacy endpoint**

- IL-6; modulation by EA-230 of inflammatory response quantified by the change in IL-6 plasma concentration over time.

**Key secondary efficacy endpoint**

- GFR; modulation by EA-230 of changes in renal function measured by plasma clearance of iohexol as the gold-standard to determine GFR.

**Other secondary efficacy endpoints**

- Inflammatory:
    - Modulation by EA-230 of the inflammatory response quantified by the change in plasma concentration over time of IL-8, IL-10, IL-17A, TNFa, IL-1RA, MCP-1, MIP1α, MIP1β, VCAM, ICAM
    - Modulation by EA-230 of the leukocyte response, quantified by change of total cell counts over time
    - Modulation by EA-230 of changes in body temperature in $^{o}$C over time
    - Modulation by EA-230 of change in SOFA score over time
    - Modulation of EA-230 of change in insulin sensitivity during IC admission/24 hrs

- General outcome:
  - Modulation by EA-230 of length of stay on ICU
  - Modulation by EA-230 of length of hospital stay
  - Modulation by EA-230 of 28 and 90-days mortality
  - Modulation by EA-230 of major clinical adverse events within 90-days (stroke, MI, rethoracotomy, readmission, pleural and/or pericardial punction)
  - Modulation by EA-230 of APACHE IV score at ICU admission
- Renal:
  - Modulation by EA-230 of GFR measured by endogenous creatinine clearance (ECC) using urine and plasma creatinine.
  - Modulation by EA-230 of GFR estimated by MDRD-calculation using plasma creatinine
  - Modulation by EA-230 of plasma creatinine and plasma proenkephalin levels
  - Modulation by EA-230 of changes in urine output
  - Modulation by EA-230 of changes in urinary renal damage markers over time of KIM-1, NGAL, L-FABP, TIMP-2*IGFBP-7, urinary IL-18, NAG
  - Modulation by EA-230 of changes in urea, sodium, creatinine and albumin in urine over time
  - Modulation by EA-230 in need for and duration of RRT
  - Modulation by EA-230 in incidence of different stages of AKI according to the RIFLE criteria
- Cardiovascular:
  - Modulation by EA-230 in vasopressor use expressed as inotropic score: (dopamine dose × 1 µg/kg/min) + (dobutamine dose × 1 µg/kg/min) + (adrenaline dose × 100 µg/kg/min) + (noradrenaline dose × 100 µg/kg/min) + (phenylephrine dose × 100 µg/kg/min) + (vasopressin (mUnits/kg/min)*10000) + (milrinone x 10mcg/kg/min)
  - Modulation by EA-230 of use of fluid therapy (ml) and fluid balance (ml)
  - Modulation by EA-230 of CK and troponine-T levels
  - Modulation by EA-230 of thoraxdrain production
  - Modulation by EA-230 of cardioplegia fluid (ml)
- Pulmonary:
  - Modulation by EA-230 of A-a $O_2$ gradient

**Pharmacokinetics:**

- Peak blood plasma levels of EA-230 10 minutes before start of extracorporeal circulation (ECC) and 10 minutes before termination of ECC
- Blood plasma levels of EA-230, AUC, Cmax, terminal $t_{1/2}$, Cl, V for a limited number of patients receiving active medication (PK population, n≈15)

## 4.6 Study Schedule

The complete schedule of prescheduled visits and all procedures to assess the above mentioned endpoints is presented in Table 1.

Radboudumc Intensive Care

QPS helps you navigate

## Tabel 1 Study Schedule

| **Study contact moments** | Pre-clinical | Pre-operative day | | | Treatment day | | | | | | | | Post-operative day | | | | Follow-up | | |
|---|---|---|---|---|---|---|---|---|---|---|---|---|---|---|---|---|---|---|---|
| **Days relative to dosing of EA-230** | -X | -X | | | 0 | | | | | | | | 1 | | | | x | 28 | 90 |
| **Timing** | | **T1** | **T2**=T1 +90 | **T3**=T1 +240 | **T4**=Pre-incision | First incision | **T5**=Start ECC | **T6**=Stop ECC | **T7**=T6 +120 | **T8**=T6 +240 | **T9**=T6 +360 | **T9A**=T6 +720 | **T10**= postop. | **T11**=T10 +90 | **T12**=T10 +180 | **T13**=T10 +240 | Hospital stay | Day 28 | Day 90 |
| Informing patient | X | | | | | | | | | | | | | | | | | | |
| Informed consent | X | | | | | | | | | | | | | | | | | | |
| In-/exclusion criteria | X | | | | | | | | | | | | | | | | | | |
| Demographics | X | | | | | | | | | | | | | | | | | | |
| Medical history | X | | | | | | | | | | | | | | | | | | |
| | | | | | | | | | | | | | | | | | | | |
| Hospitalization | | → | → | → | → | → | → | → | → | → | → | → | → | → | → | → | → | | |
| Adverse events | → | → | → | → | → | → | → | → | → | → | → | → | → | → | → | → | → | → | → |
| Vital signs[7] | | | | | | | | → | → | → | → | → | → | → | → | → | | | |
| Temperature[8] | | | | | | | | → | → | → | → | → | → | → | → | → | | | |
| Vasopressor score[8] | | | | | | | | → | → | → | → | → | → | → | → | → | X | | |
| Fluids and fluid balance[9] | | | | | | | | → | → | → | → | → | → | → | → | → | X | | |
| SOFA, A-a, cardiac enzymes | | | | | | | | | X | | | | X | | | | | | |
| Placement i.v. cannula | | X | | | | | | | | | | | | | | | | | |
| Randomization | | X | | | | | | | | | | | | | | | | | |
| IMP preparation | | | | | X | | | | | | | | | | | | | | |
| Insulin sensitivity[10] | | | | | | | | | → | | → | | → | | → | | | | |
| ***i.v. administrations*** | | | | | | | | | | | | | | | | | | | |
| EA-230 | | | | | | → | → | → | | | | | | | | | | | |
| Iohexol | | X | | | | | | | | | | | X | | | | | | |
| | | | | | | | | | | | | | | | | | | | |
| ***Sampling*** | | | | | | | | | | | | | | | | | | | |
| Iohexol[1] | | X | X | X | | | | | | | | | X | X | X | X | | | |
| Cytokines[2] | | | | | X | | X | X | X | X | X | | X | | | | | | |
| Hematology[3] | | | | | X | | X | X | X | X | X | | X | | | | | | |
| Biochemistry[4] | | X | | | | | | | | | | | X | | | | | | |
| Urine[5] | | | | | X | | | | X | X | X | | X | | | | | | |
| Pharmacokinetics[6] | | | | | | | X | X | → | → | → | | | | | | | | |
| Creat + proenkephalin | | | | | X | | | X | X | X | X | X | X | | | | X | | |

1 Renal function (GFR): Blood sampling for the determination of the renal clearance of iohexol (EDTA)
2 Cytokines: Blood sampling for the determination of Cytokines (EDTA + LH)
3 Hematology: Hb, Ht, leukocytes, thrombocytes, leukocyte differential (EDTA)
4 Biochemistry: sodium, potassium, creatinine, urea, alkaline phosphatase, ALT, AST, γGT, CK, CRP, bilirubin, glucose
5 Urine: Urine sampling for the determination kidney damage markers and endogenous creatinine clearance.
6 Pharmacokinetics: at '?'only a limited amount of patients receiving active medication are needed: n=15. Sampling times: t=0 (stop ECC), 1, 2, 5, 10, 20, 30, 60, 120, 240, 360.
7 Measured every half hour in the first 24 hours after surgery
8 Measured every 2 hours in the first 24 hours after surgery
9 Measured over a 6 hour period in the first 24 hours after surgery
10 Measured during first 24h of ICU admission at periods 0-6, 6-12, 12-18 and 18-24 hours after ICU admission.

# 5. DESCRIPTION OF INCLUDED PATIENTS

## 5.1 Analysis Populations

### 5.1.1 Intent-to treat (ITT) Population

The Intention To Treat (ITT) population includes all patients who were randomized and received study treatment, irrespective of satisfying other end point criteria. This population will be used for the analyses of safety and tolerability and all other endpoints.

### 5.1.2 Pharmacokinetic (PK) Population

The PK population will include a limited subset of approximately 15 patients for a full PK evaluation of EA-230. Because of the blinded design of the study, additional blood samples will be drawn from 30 patients during infusion of EA-230 or placebo (1:1 ratio) until 6 hours after termination of study drug administration. Patients who received EA-230, had at least one evaluable PK parameter and had not been excluded from analysis for protocol deviations or other events that impact the calculation or interpretation of the PK variables will be included in the PK subset population. As placebo and EA-230 are equally randomized, this PK population will approximately include 15 patients.

### 5.1.3 Per-Protocol (PP) Population

Analysis on the per-protocol (PP) set will be used as a supplement to the ITT analysis and will be performed for all endpoints except safety related endpoints. The PP includes all ITT patients who have not been excluded from analyses for major protocol deviations.

### 5.1.4 Strata Populations / Subgroup Populations

Strata used for the stratified randomization will also be used for subgroup analyses and all endpoints will be analyzed in these subgroups:

- CABG surgery with or without valve replacement (2 groups)
- pre-operative kidney function with an estimated GFR of ≤30, 31-90 and >90 ml/min/1,73 $m^2$ (3 groups)
- EuroSCORE II of <4 or ≥4. (2 groups)

## 5.2 Status of the patients

The status of the patients will be presented using a status dataset that will be created and used for the programming of the tables, graphs and listings. This status will contain information on the patients that received study drug and patients that completed the study. In addition, it contains the information for patients in the ITT and the PP population.

### 5.3 Protocol Deviations

A protocol deviation is any change, divergence, or departure from the study design or procedures of the study protocol. All important protocol deviations related to study inclusion or exclusion criteria, conduct of the trial, patient management or patient assessment will be collected during the conduct of the study and listed. The pre-determined categories for relevant protocol deviations are:

- Those who entered the study even though they did not satisfy the inclusion criteria
- Those who developed withdrawal criteria during the study, but were not withdrawn
- Those who were withdrawn prematurely, not completing all planned evaluations
- Those who received the wrong treatment or incorrect dose

The Protocol Deviation list is a list which presents the deviations by patient, description of actual deviation, and by type/category. The Protocol Deviation list will serve as input for the selection of patients for inclusion in each analysis population. The generation of the Protocol Deviation list is usually created by the Data Manager (with the PI), but may involve collaboration with the Biostatistician and/or the SAS programmer, and is preferably based upon a predefined set of protocol deviations that is as inclusive as possible.

### 5.4 Creation and Documentation of the PP population

Prior to DBL and unblinding, the PI gathers the clinical information necessary to make accurate decisions in creating the PP population in an objective fashion. The required information includes (but is not limited to):

1. Protocol Deviation list
2. The patients who were early withdrawals, not completing all evaluations
3. A list of adverse events with the event name, start and end times

The decisions leading to the patients and efficacy data that comprise the PP population will be documented. Any patients or data values included in the ITT, but excluded from the PP will be identified, along with the reason for exclusion. This information will be described in the core text of the Clinical Study Report, and must be listed in a Clinical Study Report appendix.

The documents describing the PP and the decisions leading to its creation will undergo a review/QC by the Biostatistician. Documentation for the PP will be signed by the PI, and as appropriate by other reviewers. The signed documentation is stored in the study file, and a copy is distributed to relevant parties involved in the efficacy analysis.

## 6. STATISTICAL ANALYSES AND REPORT SPECIFICATIONS

### 6.1 General Considerations

Continuous variables will be presented as mean, SD, SEM, median and interquartile ranges, categorical data will be presented as frequency and percentage. Appropriate rounding will be performed for the summary statistics of baseline and safety statistics: Mean, SD, SEM, median and interquartile ranges will be presented with one more decimal than the original data; Percentages will be presented with one decimal.

For Part 1 of the study, the primary endpoint is safety and tolerability; for Part 2 the primary endpoint is change in IL-6 plasma concentrations over time (determined as change in AUEC). Importantly, all data for both parts (primary, secondary and other endpoints) will be combined and analyzed together.

Concentrations of e.g. cytokines that are below the limit will be treated conservatively and set to the lower limit of quantification. Measurements of EA-230 below quantification limit (determined in the PK-study), prior to study drug administration will be set to zero.

Additional measurements (such as unscheduled or repeat assessments) will not be included in the descriptive statistics, but will be included in patient listings only.

For the calculation of baseline corrected values, baseline is defined as the last observation prior to first dosing, which corresponds to the pre-dose measurement. If this value is not present (e.g. patient is missing the baseline value or the data was not collected at that time point), the previous non-missing evaluation will be designated as the baseline value. If no baseline or previous to baseline evaluations exist, then the baseline value will be treated as missing.

Considering the stratification: Valve replacement during the CABG procedure is normally pre-determined before the procedure. However, depending on per-operative findings, the cardiac surgeon may decide to change the indication for valve or no valve replacement. Also, the EuroSCORE II stratification was calculated prior to surgery by the study team (to enable stratification) and might differ with the official calculations performed by the cardiac surgeon later on. Both the final type of surgery and EuroSCORE II will be used for total group analyses and subgroup analyses.

## 6.2 Demographic and Baseline Characteristics

Descriptive tabulations of the screening data for demographics will be produced. Demographic data at screening will be presented for the ITT, the PP, and subgroups for both the active and placebo-treated patient groups. Appropriate descriptive statistics for age, height, weight, BMI, and gender will be performed. Differences in demographic characteristics will be analyzed using a Student's *t*-test (normally distributed data) or using a Mann-Whitney *U* test (not normally distributed data). Additionally, demographic data will be listed. Age and BMI will not be calculated in SAS, but taken from the eCRF/database.

Other baseline data, such as medical history and in-and exclusion criteria will only be listed, using the ITT population.

In addition, medical history (MH) data will be coded with the latest version of the MedDRA coding system. During the clinical conduct of the study this is version 19.0. The SAS programmer or Data Manager of the study will add the coding (using the MedDRA system and SAS) to the MH descriptions extracted from the database. From the SAS dataset, an Excel sheet will be created for approval, containing the term as well as the coding information. The investigator/medical director will approve the MH coding before database lock.

## 6.3 Study Patients

The status of the patients will be given in a summary table by treatment group, as well as overall:

- the number of patients screened (available in the database)
- the number of patients included/enrolled (signed ICF)
- the number of patients randomized
- the number of patients received study drug/treated
- completed patients
- the number of patients in the ITT population
- the number of patients in the PP population
- the number of patients in the PK population
- the number of patients in the subgroup with valve surgery
- the number of patients in the subgroup without valve surgery
- the number of patients in the subgroup GFR <30 ml/min/1,73 $m^2$
- the number of patients in the subgroup GFR31-90 ml/min/1,73 $m^2$
- the number of patients in the subgroup GFR >90 ml/min/1,73 $m^2$
- the number of patients in the subgroup EuroSCORE <4
- the number of patients in the subgroup EuroSCORE ≥4

In addition, these data will be listed in a Patient Disposition listing. Reasons for discontinuation will be listed in the Study Termination listing, together with the relevant dates.

## 6.4 Analysis of Safety and Tolerability

### 6.4.1 General Considerations

The ITT population will be used for all safety analyses. Safety evaluations will be conducted throughout study conduct. All safety assessments, including, but not limited to, adverse events, clinical laboratory evaluations and vital signs, will be listed and where appropriate summarized with descriptive statistics. All data from part 1 and part 2 will be combined and analyzed as one.

For safety analyses, the descriptive statistics presented will be frequency counts (n) and percentage for qualitative variables. Quantitative variables will be summarized with sample size (n), mean ±SD, SEM, as well as median and interquartile ranges.

### 6.4.2 Safety and Tolerability Variables

The safety variables to be presented are:

- Adverse Events (AEs)
- Serious adverse events (SAEs)
- Suspected unexpected serious adverse reactions (SUSARs)
- Laboratory safety data
- Vital Signs of the patients during their ICU stay

### 6.4.3 Adverse Events

For definitions of (Serious) Adverse Events ((S)AEs) or Suspected Unexpected Serious Adverse Reactions (SUSARs) and exemptions of these definitions used in this study, please refer to the study protocol.

The AEs are tabulated by system organ class (SOC) and preferred terms (PT) within each SOC according to the MedDRA terminology list. They will be tabulated broken down by treatment. TEAEs will be summarized in descending order according to incidence of SOC and PT, using the number and percentage of patients experiencing a TEAE, as well as the number of events.

Adverse events will also be tabulated by severity and by relationship to study drug. Summary tables will be accompanied by individual patient listings.

The same tabulations and listings will be presented for SAEs and SUSARs.

(S)AE duration and onset to AE will be added in the AE listings as “dd hh:mm”. The following calculations and derivations will be used, making the most conservative judgment.

*For duration of AE*:

- Duration = stop date and time of the event minus start date and time of the event.
- If the start or stop time is unknown, then the duration will be calculated based on stop date and start date only, and +1 day is added
- If start or stop date is unknown, the duration will be missing.

*For time to onset of AE:*

- Time to onset = start date and time of the event minus date and time of preceding drug administration.
- If the start time is unknown, then the time to onset will be calculated based on start date and date of preceding drug administration only, and +1 day is added.
- If start or stop date is unknown, time to onset will be missing.

### 6.4.4 Clinical Laboratory Evaluations

Laboratory parameters to be determined per protocol are:

| **Hematology** | **Chemistry** |
|---|---|
| Hematocrit (Ht) | Sodium |
| Hemoglobin (Hb) | Potassium |
| Leukocytes | Creatinine |
| Thrombocytes | Urea |
| Leukocyte differential blood count | Alkaline phosphatase |
| | Alanine aminotransferase (ALT) |
| | Aspartate aminotransferase (AST) |
| | Gamma-glutamyl transferase (γGT) |
| | Creatine Kinase (CK) |
| | Bilirubine |
| | C-Reactive Protein (CRP) |

Listings will only present the original values, units and reference ranges as received from the laboratory.

Laboratory safety data collected according to protocol will be summarized and listed according to protocol scheme time. Where applicable, change from baseline will be calculated. In addition, the treatment effects will be investigated using a Student's *t*-test or Mann-Whitney *U* test and repeated measures 2-way ANOVA (original scale/log-transformed scale depending on distribution), if applicable. Listings will be created of the laboratory data according to protocol, including extraneous laboratory data, if applicable. Listings of all clinical laboratory data for each patient will be provided with values outside the normal ranges or abnormal indicated. Both the PI judgment, as well as the laboratory judgment will be added in the listing.

#### 6.4.5 Vital Signs

Vital signs (blood pressure and heart rate) collected according to protocol will be analyzed during ICU admission with a maximum of 24 hours. Vital signs (VS) will be summarized using descriptive statistics for each scheme time (which is, every half hour). Blood pressure and heart rate are monitored continuously. The mean value for all half-hour measurements will be calculated. Where applicable, change from baseline will be calculated. Listings of all vital sign data will be provided per half hour, including extraneous VS data collected, if applicable.

Most patients are discharged from the ICU after approximately 15 hours (namely the next morning). Therefore, the primary analysis will be conducted using the available data sets. In a second analysis 'last observation carried forward' until 24 hours after admission to ICU will be applied. The modulation by EA-230 of changes in VS will be investigated using repeated measures 2-way ANOVA.

Because of the sensitive nature of the monitoring systems, several circumstances can incorrectly influence the data. For example, movement of the hand/wrist of the patient can give incorrect blood pressure data derived from a radial artery line. Especially just before disconnecting the systems towards the discharge from the ICU, false values can be collected as the arterial line is closed of left open following removal. Therefore, we will analyse the data for irregularities and outliers using Excel's function 'conditional formatting' and a pre-determined rule. Then, to decide if an irregular value can be physiological or is incorrect, it was reviewed by an independent physician. For blood pressure, we will exclude values whenthey are >30% higher or lower than the previous values. For the last values during disconnecting of the monitoring systems, we will exclude values when they are 20% higher or lower than the previous values.

Heart rate is more variable than blood pressure, therefore no pre-determined rule to exclude data is used and no values will be excluded.

### 6.5 Analysis of Efficacy Data

The ITT and PP population, as well as the subgroup population, will be used for all efficacy analyses.

#### 6.5.1 Primary Endpoint

The following PD parameters will be calculated for IL-6: area under the effect-time curve from time 0 to time of last data point (AUEC). AUEC will be estimated using non-compartmental approaches using Phoenix WinNonlin 6.3 or higher:

Area under the effect–time curve according to the linear trapezoidal method using the IL-6 concentration-time values from time zero to time t, where t is the time of the last IL-6 concentration (Ct)

The difference in AUEC between the active and placebo group for IL-6 will be analyzed using Student's *t*-test or Mann-Whitney *U* test, depending on their distribution.

In a secondary analysis, the time course of the IL-6 concentrations between treatment groups will be compared by repeated measures two-way analysis of variance (2-way ANOVA with interaction term, on log-transformed data if data are not normally distributed).

The PD parameters will be calculated on the basis of the actual elapsed blood sampling time points. For samples whose identity is uncertain due to possible handing/labeling errors can be treated as missing at the judgment of the PD analyst and noted accordingly.

### 6.5.2 Key Secondary Efficacy Endpoint

For the key secondary endpoint, renal function, quantified as the glomerular filtration rate, measured by plasma clearance of iohexol (iGFR), will be determined. A bolus of 5mL iohexol which contains 240mg iodine per mL and 518mg iohexol per mL will be administred via a venous canulla. The total amount of iohexol present in a bolus of 5 ml is therefore 2590 mg. Following intravenous injection, iohexol is distributed in the extracellular fluid compartment. For this first 'fast phase', the Bröchner-Mortensen correction (Eq. 4) will be used. To determine the disappearance curve of iohexol in the plasma, blood will be withdrawn during the 'slow phase', twice after the first iohexol administration (T2 and T3) and trice after the second iohexol administration (T11, T12, T13). The disappearance curve will be calculated on the basis of the actual elapsed blood sampling times.

PK analyses of plasma iohexol concentration-time data will be conducted by non-compartment IV-bolus model of Phoenix WinNonlin version 6.3 or higher and the following equations:

Area under the curve (AUC, h·ng/mL)= C0/kel = (Dose/V)/kel     Eq. 1

Glomerular filtration rate (GFR, mL/min)= Dose / AUC     Eq. 2

Then the body surface area of the subjects will be calculated according to Mosteller-method. Normalization to the body surface area of 1.73 $m^2$ will be performed (Eq. 3).

Body surface area corrected GFR (mL/min)= GFR*1.73/(√((height (cm)*weight (kg))/3600))     Eq. 3

Finally, the correction of the fast phase according to Bröchner-Mortensen will be performed using the following equation (Eq. 4).

Clearance correction (mL/min/1.73$m^2$) = (0.99078 × GFR) - (0.001218 × $GFR^2$)     Eq. 4

The difference in iohexol-derived GFR between this baseline (T1-T3) and after intervention GFR (T10-T13) will be analyzed using a 2-way ANOVA (with interaction term, on log-transformed data if data are not normally distributed).

### 6.5.3 Other Secondary Efficacy Endpoints

Other secondary efficacy endpoints will be listed and tabulated per timepoint if applicable (n, mean, SD, SEM, median, interquartile ranges for continuous variables and n with percentage for categorical variables).

Treatment differences (Active treatment vs Placebo) over time for continuous data will be analyzed using a repeated measures two-way ANOVA, on log-transformed data if data is not normally distributed. The model will include treatment and time as factor variables, with time as the repeated variable and treatment*time interaction.

Treatment differences for continuous data that are not repeated over time will be analyzed using unpaired Student's *t*-test (for normally distributed data) or Mann-Whitney *U* test (for not normally distributed data).

Categorical data will be analyzed using a chi-square test. This includes the following endpoints: 28 and 90 day mortality, incidence of major clinical events, incidence of renal replacement therapy, incidence of AKI according to the RIFLE categories [1], cardioplegia fluid used and use of inotropics following surgery.

#### 6.5.3.1 Endpoint specific considerations

The SOFA score is calculated as decribed by Vincent et al. [2]. In accordance with the manual of the Dutch Intensive Care Evaluation registration, the parameter 'Glasgow Coma Scale' for post-operative patients will be scored as: 15. All other separate parameters for the calculation of the SOFA score are included in the eCRF. The creatinine-estimated GFR will be calculated using the MDRD formula [3]. The ethnicity factor of this formula will not be used.

Insulin sensitivity and plasma concentrations of glucose were added to the study protocol in a later stage (version 4.5 of the protocol). A brief summary for the handling of this data: Insulin sensitivity data will be provided as total amount of administered insulin per time period: 0-6, 6-12, 12-18 and 18-24 hours after ICU admission. The effects of EA-230 on the amount of insulin needed will be analyzed using a Student's *t*-test (normally distributed data) or Mann-Whitney *U* test (not normally distributed data) for the AUC (per period measured) and a 2-way ANOVA (interaction term, on log-transformed data if data are not normally distributed) for the complete observation duration up to 24 hrs. Glucose plasma concentrations are collected in the same time periods to check for differences between treatment groups.

Fluid therapy is labeled in the eCRF as 'Fluid IN'. Fluid balance is the difference between Fluid IN and the loss of fluids (urine production and drain production), This can be calculated from the following eCRF data:

| |
|---|
| Fluid Balance = Fluid IN – Urine production – Drain production |

Mortality data will be listed and where appropriate summarized with descriptive statistics.

Major clinical adverse events contain (as described in paragraph 4.5 study endpoints): stroke, MI, rethoracotomy, readmission, pleural and/or pericardial punction. Data can be derived from the (Serious) Adverse Events listings.

To calculate the Alveolar–arterial gradient (A–a gradient), the following formula should be used:

$$PAO2 = (FiO2 \times [Patm - PH2O]) - (PaCO2 \div R)$$

Where FiO2 is the fraction of inspired oxygen (noted in eCRF), Patm is the atmospheric pressure (760 mmHg at sea level), PH2O is the partial pressure of water (47 mmHg at 37ºC), PaCO2 is the arterial carbon dioxide tension (noted in eCRF), and R is the respiratory quotient (determined as 0.8).

### 6.6 Analyses of pharmacokinetics of EA-230

For every patient, peak EA-230 concentration will be sampled at T5 and T6. Approximately 15 patients will be involved in PK evaluations. Blood samples of EA-230 will be collected at: T=0 (directly prior to termination of EA-230 administration), 1, 2, 5, 10, 20, 30, 60, 120, 240 and 360 minutes after termination of administration.

The PK parameters will be calculated on the basis of the actual elapsed blood sampling time points. The following plasma pharmacokinetic (PK) parameters of EA-230 will be derived by non-compartmental analysis of the plasma concentration-time profiles using Phoenix WinNonlin, version 6.3 or higher: area under the plasma concentration time curve (AUC) from time 0 to the time of the last concentration (AUC0-last), AUC from time 0 extrapolated to infinity (AUC0-inf), maximum observed plasma concentration (Cmax), time at which Cmax occurred (Tmax), apparent terminal elimination half life (t1/2), clearance after extravascular administration (Cl) and volume of distribution based on the terminal phase (Vz). The definition for each PK parameter is listed in the following table.

| | |
|---|---|
| Cmax | Maximum observed concentration |
| AUC0-last | Area under the concentration-time curve calculated using linear up/log down trapezoidal summation from time zero to time t, where t is the time of the last concentration above the limit of quantification |
| AUC0-inf | Area under the concentration-time curve from time zero to infinity, AUC0-inf = AUC0-last + %AUCextra = AUC0-last + Ct/ kel, where kel is the terminal elimination rate constant |
| kel | Apparent terminal elimination rate constant calculated by linear regression of the terminal linear portion of the log concentration vs. time curve |
| Tmax | Time of the maximum concentration |
| t1/2 | Apparent terminal elimination half-life calculated as ln(2)/ kel |
| Cl | Clearance after IV administration calculated as [Dose/ AUC0-inf] |
| Vz | Volume of distribution based on the terminal phase calculated as [Dose/(AUC0-inf * kel)] |

The measured individual plasma concentrations of EA-230 will be used to directly obtain Cmax. AUC0-t (the area under the concentration-time curve from time zero to the time of the last measurable concentration (tlast)) will be calculated using the linear up/log down trapezoidal method.

Estimates of half-life (t1/2) will be calculated using the following (Equation 1):

$$T1/2 = \ln(2)/kel \qquad \text{Eq. 1}$$

where the value of the terminal elimination rate constant (kel) will be determined by a non-compartmental analysis using WinNonlin.

A regression analysis will be performed on the terminal linear phase of the semi-logarithmic plots of individual plasma concentration time data. During the analysis, WinNonlin will repeat regressions using the last three points with non-zero concentrations, then the last four points, and the last five, etc. Points prior to Cmax and at Cmax will not be used. Points with a value of zero for the dependent variable will be excluded. For each regression, an adjusted $R^2$ is computed:

$$\text{Adjusted } R^2=1-(1- R^2)*(n-1)/(n-2) \qquad \text{Eq. 2}$$

Where n is the number of data points in the regression and $R^2$ is the square of the correlation coefficient. WinNonlin estimates kel using the regression with the largest adjusted $R^2$ and, if the adjusted $R^2$ does not improve, but is within 0.0001 of the largest adjusted $R^2$ value, the regression with the larger number of points is used. Kel must be positive and calculated from at least three data points and adjusted $R^2$ value must be not less than 0.80.

If the terminal phase for any individual subject fails to meet the stated criteria, the t1/2 will be considered to be not reportable (NR). If an estimated half-life (t1/2) is greater than tlast (the last PK blood sampling time), then, too, the half-life value will be considered to be not reportable.

AUC0-inf will be computed using the following (Equation 3):

$$AUC0 – inf = AUC0 – last + Ct/kel \qquad \text{Eq. 3}$$

where Ct is the last concentration.

If %AUCextra is greater than 20% of AUC0-inf, then t1/2, kel, AUC0-inf, CL, and Vz will not be reported and not be used in statistical analyses including descriptive statistics.

### 6.6.1 PK Data Handling Procedures

Plasma concentrations at timepoint T5 and T6 will be summarized and included in the descriptive statistics for all patients in the the PP population.

Patients included in the PK subgroup will be used for full pharmacokinetic analyses.

The data for patients with significant protocol deviations and/or violations will be cautiously evaluated, including PK sampling outside of time window. If the deviation or violation is thought to have a significant impact on the PK profile, the data for those patients should be excluded from the

study's statistical evaluation. However, the concentration data and PK parameters derived for those patients will be determined and reported.

*BQL/Pre-dose Values/Missing Values:*

Plasma concentrations below quantifiable limits (BQL) will be imputed as the lower limit of quantification (LLOQ) for the calculation of PK metrics. However, BQL of the pre-dose concentration will be imputed as zero. For unexpected BQL samples (i.e. those that come between two non-BQL samples) or samples whose identity is uncertain due to possible handing/labeling errors can be treated as missing at the judgment of the PK analyst and noted accordingly.

*AUC0-inf:*

AUC0-inf is considered reportable if the following criteria are met:

- t1/2 is estimable according to **Fout! Verwijzingsbron niet gevonden.**
- AUC0-last /AUC0-inf ratio $\geq$ 0.80.
- The adjusted $R^2$ value $\geq$ 0.80

If AUC0-inf for an individual subject fails any of the above criteria, the value will be reported as NE or NR, where

- NE: could not be estimated.
- NR: AUC0-last /AUC0-inf ratio < 0.80; therefore, t1/2, kel, AUC0-inf, CL, and Vz will not reportable.

*Data Format (significant figures and decimal points):*

Pharmacokinetic parameters will be reported to 3 significant figures for individual parameters and summary statistics, with the exception of Tmax (2 decimal places), and CV% (1 decimal place) and N which will be whole numbers (0 decimal places).

### 6.6.2 Statistical Evaluations of PK Parameters

Data from patients providing sufficient information to calculate pharmacokinetic parameters for subjects who completed the treatment will be included in the pharmacokinetic analysis. Descriptive statistics (number of patients, mean, SD, SEM, CV%, median, interquartile ranges, artimetic mean and 95% confidence intervals) for plasma concentrations of EA-230 each scheduled time point will be summarized.

Individual patient listings will be provided. Mean and individual plasma concentration-time profiles for EA-230 will be presented graphically for each treatment.

## 7. CHANGES FROM PROTOCOL AND OTHER REMARKS

There are no significant changes to the analyses planned in the study protocol.

# 8. SOFTWARE

## 8.1 Coding Systems

Adverse events and Medical History will be coded using MeDRA version 19.0.

## 8.2 Statistical Software

The statistical analysis and reporting will be done using SAS® for Windows™ version 9.3 or higher SAS output will be saved and imported into Microsoft Office Word®.

## 8.3 Reporting

All safety output will be generated as SAS tables and listings. All tables and listings will be created such that they fit landscape pages. All tables and listings will be created using SAS with an RTF output, and font Calibri size 10 will be used.

A list of tables, graphs and listings is presented (per report section) in Appendix A.

The QPS template tables and listings will be used, and a separate templates document will be supplied together with the SAP. Adaptations to template layout are possible depending on the design of the study, the length of variables and the number of variables. It should be noted that all data as collected will be presented in listings and/or tabulations. The examples in the templates document may not cover all possible collected data, or examples may be present of data not collected for this specific study.

All tables and listings created will need to adhere to the following margins to fit the appendix layout if the CSR:

Portrait: Top - 1.0 inch
Bottom - 1.0 inch
Left - 1.25 inch
Right - 1.0 inch

Landscape: Top - 1.25 inch
Bottom - 1.0 inch
Left - 1.0 inch
Right - 1.0 inch

# 9. TABLES, LISTINGS AND FIGURES

All tables and graphs mentioned here will be presented in report, and will be supplied to the Medical Writer as separate .RTF and .docx files.

- Summary table of demographic data, including weight, height and BMI at screening, for ITT, PP, PK population and strata populations: Mean, SD, SEM, median, interquartile ranges, for quantitative variables. Frequency and percentage for qualitative variables.

- Summary table for primary efficacy endpoints per treatment: Mean, SD and SEM, or Median and inter quartile ranges.

- Adverse event summary containing the number and percentage of patients experiencing any AE or SAE or SUSAR. Table will contain information concerning the severity and relationship, concomitant medication given and discontinuation due to an AE. Table will be presented by treatment group.

- Adverse event summary containing the number and percentage of patients experiencing treatment-emergent AEs. AEs are tabulated by MedDRA SOC and preferred term, and summarized by treatment group. AEs will be summarized in descending order according to incidence of SOC and preferred term.

- If applicable, the same tabulations will be presented for SAEs.

- If applicable, the same tabulations will be presented for SUSARs.

- If applicable: summary listing of serious adverse events containing description of event, MedDRA preferred term, patient number, relationship, action taken and outcome.

- Summary tables presenting the results of the primary analyses for the **primary endpoints**.

- Summary tables presenting the results of the analyses for the **secondary endpoints**.

- Summary tables presenting the results of the **subgroup analyses** for the all endpoints.

## 9.1 List of Tables and Figures

All tables and graphs mentioned here will be presented according to ICH guidelines in appendix 14 of the report. A complete document ('batch load') will be created in Word for the Medical Writer, in the order and with section number and title as stated.

| Section | Title | Notes |
|---|---|---|
| 14.1 | Summary patient disposition | Summary of all randomized/enrolled and completed patients per treatment, and the number of patients in the ITT, PP, PK population as well as strata populations |
| 14.2 | Summary Demographic data | Descriptive statistics for demographic data For ITT, PP and PK population as well as strata populations |
| 14.3 | Student's t-test/Mann-Whitney U test of Weight, Height and BMI | Student's *t*-test or Mann-Whitney *U* test |
| 14.4<br>14.5.1<br>14.5.2<br>14.6.1<br>14.6.2<br>14.7.1<br>14.7.2<br>14.8 | Descriptive Statistics of Inflammatory Endpoints<br>Descriptive Statistics of General Outcome (Continuous Variables)<br>Descriptive Statistics of General Outcome (Categorical Variables)<br>Descriptive Statistics of Renal Endpoints (Continuous Variables)<br>Descriptive Statistics of Renal Endpoints (Categorical Variables)<br>Descriptive Statistics of Cardiovascular Endpoints (Continuous Variable)<br>Descriptive Statistics of Cardiovascular Endpoints (Categorical Variable)<br>Descriptive Statistics of Pulmonary Endpoints | Descriptive statistics for absolute values and change from baseline by time point, per treatment for continuous variables.<br>Discriptive statistics for categorical variables: mortality, major clinical events, Need of RRT, AKI (RIFLE), Cardioplegia fluid, inotropics given on day 0-7. |
| 14.9<br>14.10<br><br>14.11<br><br>14.12<br>14.13<br>14.14<br>14.15 | Subjects Excluded from PK or IL-6 AUEC or iGFR Analyses<br>Summary of Pharmacokinetic Parameters of EA-230 for Subjects with Intensive PK samples<br>Summary of Pharmacokinetic Parameters of EA-230 for Subjects with Sparse PK samples<br>Summary of IL-6 AUEC<br>Summary of iGFR (ml/min/1.73m$^2$)<br>Repeated Measures ANOVA for iGFR (ml/min/1.73m$^2$)<br>Repeated Measures ANOVA for IL-6 Concentration | Summary tables of pharmacokinetic and pharmaodynamic analyses<br>Repeated Measures ANOVA for iGFR (GFR measured by plasma clearance of iohexol) and IL-6 Concentration |

| Section | Title | Notes |
|---|---|---|
| 14.16<br>14.17<br>14.18 | Repeated Measures ANOVA for Hematology endpoints<br>Repeated Measures ANOVA for Biochemistry<br>Repeated Measures ANOVA for Vital signs | For laboratory and vital signs parameters RM 2-way ANOVA will be performed |
| 14.19<br>14.20<br>14.21<br>14.22<br>14.23<br>14.24 | Student's t-test or Mann–Whitney U test for IL-6 (AUEC)<br>Student's t-test or Mann–Whitney U test for Inflammatory endpoints<br>Student's t-test or Mann–Whitney U test for General Outcome<br>Student's t-test or Mann–Whitney U test for Renal endpoints<br>Student's t-test or Mann–Whitney U test for Cardiovascular endpoints<br>Student's t-test or Mann–Whitney U test for Pulmonary endpoints | Student's *t*-test or Mann–Whitney *U* test for IL-6 (AUEC), insulin sensitivity, LOS ICU, LOS H, APACHE IV, GFR (ECC), plasma reatinine day 0-7, days of RRT, fluid balance day 0 - 7 |
| 14.25<br>14.26<br>14.27<br>14.28 | Repeated Measures ANOVA for Inflammatory endpoints<br>Repeated Measures ANOVA for Renal endpoints<br>Repeated Measures ANOVA for Cardiovascular endpoints<br>Repeated Measures ANOVA for Pulmonary endpoints | Repeated Measures 2-way ANOVA (original scale/log-transformed scale depending on distribution) for cytokines, leukocytes, body temperature, insulin sensitivity, SOFA score, eGFR (MDRD), plasma creatinine, plasma proenkephalin, urine output, urine damage markers, urine biochemistry, A-a $0_2$ gradient, Vasopressor score, fluid therapy, fluid balance, CK, Troponin-t, thoraxdrain production |
| 14.29<br>14.30<br>14.31 | Chi-Squared Test for General Outcome<br>Chi-Squared Test for Renal Endpoints<br>Chi-Squared Test for Cardiovascular Endpoints | Chi-Squared test for categorical variables: mortality, major clinical adverse events, need of RRT, AKI (RIFLE), cardioplegia fluid, inotropics given day 0-7 |
| 14.32.1 | Summary Adverse Events | Adverse event summary containing the number and percentage of patients experiencing any AE or SAE or SUSAR. Table will contain information on the severity of the AE, whether concomitant medication was given, whether the AE resulted in discontinuation from the study and relationship |

Radboudumc Intensive Care

QPS helps you navigate

| Section | Title | Notes |
|---|---|---|
| | | to the study drug. Table will be presented per treatment. |
| 14.32.2 | Summary Adverse Events by System Organ Class, Preferred Term | Adverse event summary containing the number and percentage of patients experiencing AEs. AEs are tabulated by SOC and PT, and summarized by treatment. The summary will be presented in descending according to SOC and preferred term. |
| 14.32.3 | Summary Adverse Events by System Organ Class, Preferred Term and Severity | The same tabulation as 14.3.1.2 will be created for AEs by severity . |
| 14.32.4 | Summary Adverse Events by System Organ Class, Preferred Term and Relationship | The same tabulation as 14.3.1.2 will be created for AEs by relationship to study drug. |
| 14.33.1<br>14.33.2<br>14.33.3<br><br>14.33.4 | Summary Serious Adverse Events<br>Summary Serious Adverse Events by System Organ Class, Preferred Term<br>Summary Serious Adverse Events by System Organ Class, Preferred Term and Severity<br>Summary Serious Adverse Events by System Organ Class, Preferred Term and Relationship | If applicable, the same tabulations as 14.34.1 to 14.34.4 will be presented for SAEs. |
| 14.34.1<br>14.34.2<br>14.34.3<br>14.34.4 | Summary SUSARs<br>Summary SUSARs by System Organ Class, Preferred Term<br>Summary SUSARs by System Organ Class, Preferred Term and Severity<br>Summary SUSARs by System Organ Class, Preferred Term and Relationship | If applicable, the same tabulations as 14.34.1 to 14.34.4 will be presented for SUSARs. |
| 14.35 | Out of range clinical laboratory values | A patient listing of all data outside the (investigators) reference range, containing the variables test, gender, age, date/time, value, reference range, and whether there were clinical implications (CI/NCI). |

| Section | Title | Notes |
|---|---|---|
| 14.36<br>14.37 | Summary Clinical Laboratory data for Hematology<br>Summary Clinical Laboratory data for Biochemistry | Descriptive statistics for absolute values and change from baseline by time point, per treatment for Hematology and Biochemistry. |
| 14.38 | Summary Vital Signs data | Descriptive statistics for absolute values and change from baseline by time point, per treatment. |
| Figure 14.1<br>Figure 14.2<br>Figure 14.3<br>Figure 14.4 | IL-6 Plasma Concentration (pg/ml) Mean (with SEM)<br>EA-230 Plasma Concentration Mean (with SEM)<br>Iohexol Plasma Concentrations Mean (with SEM) – Day -1<br>Iohexol Plasma Concentrations Mean (with SEM) – Day +1 | Mean concentration figures of Il-6, EA-230 and Iohexol. |

## 9.2 List of Patient Data Listings

All listings mentioned here will be presented according to ICH guidelines in appendix 16.2 of the report. A complete document ('batch load') will be created in Word for the Medical Writer, in the order and with section number and title as stated.

Individual listings will be prepared of all the data collected in the database. No combining of data other than mentioned in this paragraph will be performed. Listings will be presented per treatment. The key variables in all listings will be patient number and treatment. If applicable, period/visit number, day and time point will be listed additionally. For laboratory data, gender and age will be added to the listing, if deemed relevant. For AE data, duration and time to onset will be added.

Additionally, a listing containing study dates and times will be presented, as well as containing PK concentration data and PK sampling time deviations.

For inclusion/exclusion criteria, the text from the core protocol will be used (not the text from the synopsis or the source/eCRF) for listing purposes.

The individual and descriptive statistics of subject plasma EA-230 concentrations and pharmacokinetic parameters will be listed. Individual subject and descriptive statistics for plasma IL-6 concentrations and IL-6 AUEC will be included. The individual subject plots of plasma

concentration vs. time of EA-230, iohexol and the IL-6 will be demonstrated. WinNonlin outputs will be included in listing. Statistical analysis results of GFR and IL-6 AUEC will be listed.

| Section | Title | Notes |
|---|---|---|
| 16.2.1.1 | Compliance to inclusion and exclusion criteria | Compliance to inclusion and exclusion criteria |
| 16.2.1.2 | Listing patient disposition (All patients population) | Listing patient disposition. Including ITT, PP and PK population |
| 16.2.1.3 | Listing study termination | Listing study termination |
| 16.2.2.1<br>16.2.2.2 | Pharmacokinetics blood sampling time deviations<br>Other blood sampling time deviations | Blood sampling time deviations |
| 16.2.2.3 | Protocol deviations | Protocol deviations |
| 16.2.4.1 | Listing demographic data at screening | Listing demographic data at screening.Including Height, Weight, BMI |
| 16.2.4.2 | Medical history | Medical history. Including MedDRA coding |
| 16.2.5.1 | Listing Drug administration | Listing dose administration |
| 16.2.5.2 | Listing Pharmacokinetics blood sampling dates and times | Listing blood sampling dates and times. Including scheduled and actual scheme times |
| 16.2.5.3.1<br>16.2.5.3.2 | Listing study dates and times<br>Listing study dates and times – continued | Listing study dates and times |
| 16.2.6.6<br>16.2.6.7<br>16.2.6.7.1 | Individual Subject EA-230 Concentrations<br>Individual and Descriptive Statistics of EA-230 Pharmacokinetic Parameters<br>Individual and Descriptive Statistics of EA-230 Pharmacokinetic Parameters for Subjects with Intensive PK Samples<br>Individual and Descriptive Statistics of EA-230 (Cmax) Subjects with Sparse PK | Individual and descriptive pharmacokinetic , pharmacodynamic and GFR data |

| Section | Title | Notes |
|---|---|---|
| 16.2.6.7.2<br><br>16.2.6.8<br>16.2.6.8.1<br><br>16.2.6.8.2<br><br>16.2.6.9<br>16.2.6.9.1<br>16.2.6.9.2<br>16.2.6.10<br><br>16.2.6.10.1<br><br>16.2.6.10.2<br><br>16.2.6.11<br>16.2.6.12<br>16.2.6.13<br>16.2.6.14 | Samples<br>Individual and Descriptive Statistics of Plasma Concentration of Cytokine IL-6<br>Individual and Descriptive Statistics of Plasma Concentration of Cytokine IL-6 after IV Infusion of EA-230<br>Individual and Descriptive Statistics of IL-6 Plasma Concentration after IV Infusion of placebo<br>Individual and Descriptive Statistics of AUEC of IL-6<br>Individual and Descriptive Statistics of AUEC of IL-6 after IV Infusion of EA-230<br>Individual and Descriptive Statistics of AUEC of IL-6 after IV Infusion of Placebo<br>Individual and Descriptive Statistics of Iohexol Plasma Concentrations and GFR<br>Individual and Descriptive Statistics of Iohexol Plasma Concentrations and GFR for subjects with IV Infusion of EA-230<br>Individual and Descriptive Statistics of Plasma Concentrations and GFR for Subjects with Placebo<br>Individual Subject EA-230 Plasma Concentration-Time Graphs<br>Individual Subject IL-6 Plasma Concentration-Time Graphs<br>Individual Subject Iohexol Plasma Concentration-Time Graphs<br>WinNonlin Output | |
| 16.2.7.1.1<br>16.2.7.1.2<br>16.2.7.1.3 | Listing Adverse Events<br>Listing Adverse Events – continue 1<br>Listing Adverse Events – continue 2 | Adverse events. Including MedDRA coding |
| 16.2.8.1<br>16.2.8.2 | Listing laboratory results <Chemistry/Hematology - quantitative results><br>Listing laboratory - comments | Listing Laboratory safety data. Including extraneous data and comments |
| 16.2.9 | Listing Vital signs | Listing Vital signs |
| 16.2.10 | Listing prior and concomitant medication | Listing prior and concomitant medication |
